# A deformability-based biochip for precise label-free stratification of metastatic subtypes using deep learning

**DOI:** 10.1038/s41378-023-00577-1

**Published:** 2023-09-28

**Authors:** Haojun Hua, Shangjie Zou, Zhiqiang Ma, Wang Guo, Ching Yin Fong, Bee Luan Khoo

**Affiliations:** 1 City University of Hong Kong, 83 Tat Chee Avenue, Kowloon, Hong Kong, 999077 China; 2 Hong Kong Center for Cerebro-Cardiovascular Health Engineering (COCHE), Hong Kong, 999077 China; 3 City University of Hong Kong Futian-Shenzhen Research Institute, Shenzhen, 518057 China

**Keywords:** Microfluidics, Bionanoelectronics

## Abstract

Cellular deformability is a promising biomarker for evaluating the physiological state of cells in medical applications. Microfluidics has emerged as a powerful technique for measuring cellular deformability. However, existing microfluidic-based assays for measuring cellular deformability rely heavily on image analysis, which can limit their scalability for high-throughput applications. Here, we develop a parallel constriction-based microfluidic flow cytometry device and an integrated computational framework (ATMQcD). The ATMQcD framework includes automatic training set generation, multiple object tracking, segmentation, and cellular deformability quantification. The system was validated using cancer cell lines of varying metastatic potential, achieving a classification accuracy of 92.4% for invasiveness assessment and stratifying cancer cells before and after hypoxia treatment. The ATMQcD system also demonstrated excellent performance in distinguishing cancer cells from leukocytes (accuracy = 89.5%). We developed a mechanical model based on power-law rheology to quantify stiffness, which was fitted with measured data directly. The model evaluated metastatic potentials for multiple cancer types and mixed cell populations, even under real-world clinical conditions. Our study presents a highly robust and transferable computational framework for multiobject tracking and deformation measurement tasks in microfluidics. We believe that this platform has the potential to pave the way for high-throughput analysis in clinical applications, providing a powerful tool for evaluating cellular deformability and assessing the physiological state of cells.

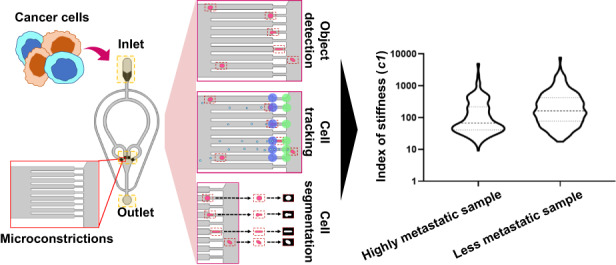

## Introduction

Precision medicine is highly sought after due to the extreme heterogeneity of cancer tumors^1^. However, current diagnostic methods predominantly rely on costly label-based analysis techniques, necessitating complex procedures^2^. Label-free techniques based on intrinsic properties reflecting key cancer progression events such as metastasis are a promising alternative to marker-based assays in developing countries.

Structural features, such as cytoskeletal composition, nuclear density, and chromatin texture, can directly reflect the physiological state of cells. Cellular deformability has been linked to several medical conditions, including malaria^3^, sepsis^4^, bacterial infection^5^, and cardiomyocyte pathology^6^. Additionally, the ability of cancer cells to metastasize has been linked to their deformability^7,8^, and a correlation between cellular deformability and extravasation through the vessel wall has been reported^9^. Therefore, cellular deformability is a promising label-free biomarker for assessing cancer cell metastatic potential and enabling simple, low-cost diagnostic assays for precision medicine.

The correlation between cellular deformability and disease progression has led to the development of several high-throughput deformability-based cytometry systems using microfluidics^10^. Some models use electrical-based detection to quantify deformability^11^. Such technologies have achieved high processing throughput^12^, presenting great potential in label-free biosensing due to their capability to acquire multiple biophysical signals^13^. Despite these advantages, image-based detection methods remain prevalent due to their ease of fabrication and maintenance. However, balancing device cost, throughput, and pathological relevance is challenging in image-based microfluidics.

Shear flow deformability cytometry (sDC) and extensional flow deformability cytometry (xDC) have very high throughput^14,15^. However, these technologies require high-speed cameras capable of operating at excessively high frame rates, often exceeding 10,000 frames per second, resulting in prohibitively high costs, particularly in developing countries. sDC and xDC are also insensitive to cellular friction and retention changes, which are crucial for determining cancer cell metastatic potential^10^. On the other hand, constriction-based deformability cytometry (cDC) requires much lower imaging frame rates^16^ and remains sensitive to changes in metastatic potential. Nonetheless, most existing cDC systems have low throughput.

Here, we present the development of a constriction-based deformability cytometry (cDC) platform to evaluate the metastatic potential of cancer cells. The cDC platform is a sensitive, high-throughput, low-cost method that can provide quantitative readouts of cell friction and retention at approximately 25,000 cells per minute. To enable high-throughput analysis while remaining cost-effective, we developed a deep learning-based computational framework called ATMQcD, which includes modules for automatic training set generation, multiobject tracking, segmentation, and quantification of cellular deformability. Our cDC device and ATMQcD computational framework outperformed previous technologies in terms of analytical throughput while being more sensitive and cost-effective (Supplementary Table 1).

Our study represents the first to combine high-throughput cDC microfluidic chips with advanced deep-learning algorithms to assess cancer metastasis potential. The cDC + ATMQcD system, which utilizes our cell stiffness index (c1 index), can evaluate the invasiveness of clinically relevant heterogeneous cancer cell populations and cancer cells from multiple cancer types, including breast cancer, lung cancer, and urinary bladder cancer, at the single-cell level. We found that the cDC + ATMQcD system outperformed conventional methods for metastatic evaluation in terms of efficiency, sensitivity, and cost-effectiveness (Supplementary Table 2). We envision that the cDC + ATMQcD system has the potential to be a complementary and promising tool for rapid screening and precision clinical diagnosis in global health care settings.

## Result

### Development of a constriction-based deformability cytometer (cDC) with high sensitivity for assessing cancer cell metastasis

**Fig. 1:**
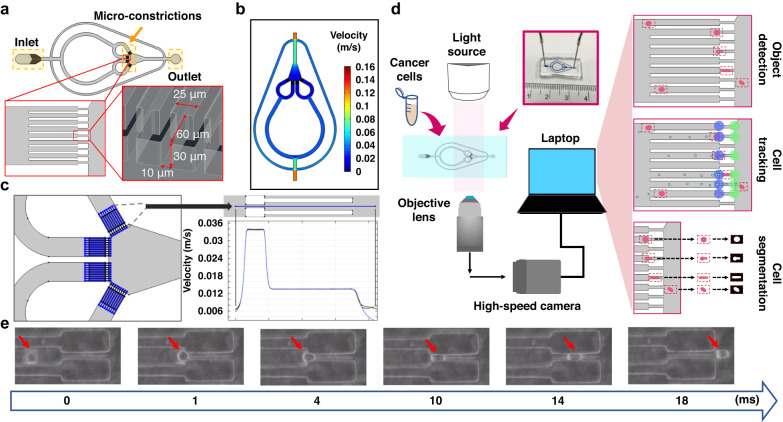
Design of the constriction-based deformability cytometry (cDC) platform. **a** Schematic diagram of the microfluidic device. The device had one inlet, one outlet, and four groups of microconstrictions. Samples were introduced through the device inlet and deformed in the narrow microconstrictions. **b** Top view of laminar flow velocity simulation of the microfluidic chip under a 50 μL/min flow rate. **c** The fluid flow velocity across the 36 microconstrictions under a 50 μL/min flow rate. The blue lines in the left subplot demonstrate the measuring lines in the COMSOL simulation. A scaled subplot showing a measuring line crossing a microconstriction (upper right). A line plot (bottom-right) displays velocity across the 36 microconstrictions denoted in the left subplot. **d** Schematic view of the cDC platform’s experimental setup and the computational framework for automatic training set generation, multiple object tracking, segmentation, and cellular deformability quantification (ATMQcD). **e** Time-lapse imaging demonstrated the cell deformation and movement process while passing through a microconstriction

In pursuit of an efficient and cost-effective means of measuring cell mechanical properties with high sensitivity and throughput, we developed a parallelized cDC (constriction-deformability cytometry) device that features four groups of microconstrictions, each containing nine individual microconstrictions (Fig. 1a). The design of our microfluidic device allowed us to process samples with high throughput and a wide frame of view, enabling us to achieve a notable improvement in efficiency.

Before entering the microconstrictions, individual cells were separated using narrow channels with 25 μm widths (Fig. 1a) to prevent interference between them. The cancer cell size is heterogeneous, ranging from 13 to 25 μm^17^. Here, each microconstriction measured 10 × 30 × 60 μm (width × height × length), with a width at least 2 μm narrower than most cancer cells. As a result, cells were forced to deform when passing through these microconstrictions. Bypass valves maintained constant pressure drops between the inlet and outlet. Our device was fabricated using photolithography and soft lithography techniques following established protocols^18,19^.

To showcase the versatility of our cDC device in simultaneously measuring fluidic-dependent parameters, such as motility parameters, in multiple microconstrictions, we conducted a velocity simulation in COMSOL. In our study, we employed an inlet velocity of 0.08998 m/s, which was derived from experimental findings indicating an optimal flow rate of 50 μL/min (see "Optimization of the cDC platform for single-cell analysis" for details) (Fig. 1b). Our results indicated that the velocity remained nearly constant across all 36 microconstrictions, suggesting the absence of any systematic differences in fluidic properties (Fig. 1c).

To determine the maximum throughput of the cDC device, we conducted an experiment in which we introduced a very high concentration of cells (2 million cells per mL) into the device at a flow rate of 50 μL/min. We captured multiple videos over several minutes until severe microconstriction-related device fouling occurred and analyzed the average number of cells detected per second using the ATMQcD framework for each video. Our results revealed that the cDC+ATMQcD system successfully detected over 700 cells per second at its peak, with an average detection efficiency of approximately 385 cells per second (Supplementary Fig. 1). This implies that the cDC device could process approximately 20,000 to 25,000 cells per minute.

However, it is important to note that achieving such a high throughput is challenging due to the need for high cell concentrations, which increases the risk of microconstriction-related device fouling. In practice, we typically employ lower cell concentrations and high-magnification microscopic imaging, focusing on a single group of microconstrictions, to enhance detection sensitivity (section “Optimization of the cDC platform for single-cell analysis”).

### Optimization of the cDC platform for single-cell analysis

We optimized several sample-related settings on the cDC platform, including the infusion flow rate and cell concentration, to optimize the robust platform with clinically relevant readouts.

Cells were infused into the microfluidic chip using a syringe pump during sample processing. A high-speed camera attached to a microscope captured videos of the samples, which were then saved on a laptop for further analysis (Fig. 1d). We evaluated the infusion of cell samples into the cDC device at three different flow rates: 30 μL/min, 50 μL/min, and 70 μL/min. At a flow rate of 30 μL/min, we observed biofouling, likely due to insufficient fluidic pressure (Supplementary Fig. 2a). Conversely, at a flow rate of 70 μL/min, the high pressure caused cells to move too quickly, resulting in reduced imaging resolution and readout accuracy (Supplementary Fig. 2a). Consequently, we fixed the flow rate at 50 μL/min for samples with a typical cell size range (12–16 μm) for subsequent analysis (Fig. 1e).

Furthermore, we evaluated the optimal concentration for the platform. Our results indicated that low cell concentrations (e.g., <2.5 × 10^5^ cells/mL) led to reduced throughput (Supplementary Fig. 2b), while higher concentrations (>1 × 10^6^ cells/mL) had the potential to cause cell aggregation and biofouling after the system had been running for over 5 min (Supplementary Fig. 2b). Consequently, we fixed the cell concentration at 5 × 10^5^ cells/mL for subsequent analysis (a representative timelapse showing the optimal flow conditions in the cDC device is shown in Supplementary Video 1).

In addition to flow rates and cell concentrations, imaging settings play a crucial role in the performance of our system. Our device has been specifically designed to be compatible with microscopes equipped with two different magnifications of objective lenses. The first is a 10× objective lens that provides high-resolution images of a single group of microconstrictions, which consists of 9 microconstrictions (Supplementary Fig. 3a). The second is a 4× objective lens that allows for the capture of all 36 microconstriction channels within the same field of view (Supplementary Fig. 3b).

To determine which magnification performs better in deformability measurements, we tested two breast cancer cell lines, MCF7 and MDA-MB-231, using 4× and 10× objective lenses for imaging. The captured images were subsequently analyzed using the ATMQcD system.

Previous studies have shown that MDA-MB-231 cells are more invasive and deformable than MCF7 cells^7^. Therefore, we expected MDA-MB-231 cells to pass through the microconstrictions in a shorter time compared to MCF7 cells. However, due to the limitations of the 4× objective lens, the cell boundaries were not in sharp focus, posing challenges for cell segmentation and resulting in lower statistical significance in the unpaired t-test compared to the results obtained with the 10× objective lens (Supplementary Fig. 3c). This indicated reduced sensitivity in cell classification. As a result, for most of our experiments, we utilized the 10× objective lens for a device with 36 microconstrictions. This allowed us to capture nine microconstriction channels within a single field of view.

It is important to note that the maximum throughput of the cDC+ATMQcD system can be achieved with the appropriate setup using a combination of a 4× objective lens with high-resolution imaging equipment. Moreover, increasing the number of microconstriction areas can enhance the system’s tolerance to fouling by providing sufficient alternative microconstrictions and reducing the effects of pressure fluctuations.

### ATMQcD framework for rapid multivariate analysis

To enable clinical applications, high-throughput probing of cells using realistic samples from liquid biopsies is pivotal, which requires faster cell movement and greater detection efficiency. Therefore, developing an image analysis system capable of tracking multiple fast-moving objects simultaneously is critical.

**Fig. 2:**
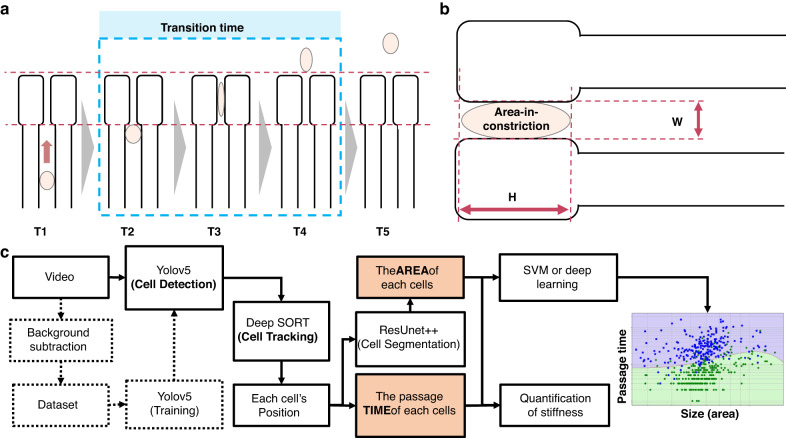
Quantification of cellular deformability and the framework of the ATMQcD platform. **a** Demonstration of passage time for a single cell. Passage time is calculated by T4-T2. **b** Demonstration of the deformation index and area-in-constriction for a single cell. The deformation index is calculated by (H − W)/(H + W). **c** The framework of the ATMQcD computational platform

Here, we utilized the passage time, area-in-constriction, and deformation index as key measurements for our deformability-based platform. Passage time refers to the time it takes for a cell to pass through a microconstriction (Fig. 2a).

As shown in Supplementary Fig. 4a, after entering a microconstriction, a cell is squeezed and deformed by the wall. The pressure of the wall is ${{\rm{F}}}_{{\rm{wall}}}$, and when the cell enters, the friction of the wall is ${\rm{friction}}={\rm{\mu }}\cdot {{\rm{F}}}_{{\rm{wall}}}$, where μ is the friction coefficient of the microconstriction. In this study, we considered the cell to be incompressible during the relatively short entry time and applied the parameter ${{\rm{\varepsilon }}}_{{\rm{e}}}$ to quantify the maximum deformation rate of cells following the formula proposed in^16^:

1 $${\varepsilon }_{e}=\frac{R-{R}_{e}}{R}$$

where $R$ is the radius of the cell and ${R}_{e}$ is half the width of the microconstriction.

Once the cells enter the microconstriction, as depicted in Supplementary Fig. 4b, a noticeable pressure difference arises, with more significant pressure on the left than on the right. This pressure difference, denoted ΔP, results in a horizontal force exerted from left to right. Previous studies have highlighted that cell passage through microconstriction can be divided into two distinct stages: creep and transit stages^20,21^. In line with these findings, we have also employed this approach to analyze the cell passage process, distinguishing between the creep and transit stages. Specifically, the creep stage is defined as the period from the moment the cell is detected at the entrance of the microconstriction until it is entirely inside the microconstriction.

In our analysis, we consider the cell to be a viscoelastic homogeneous entity. As a result, we employ power-law rheology to describe the variations in cell strain (ε) during the creeping stage (${{\rm{t}}}_{{\rm{creep}}}$). The relationship between ε and ${{\rm{t}}}_{{\rm{creep}}}$. is described by Eq. (2)^16,22,23^, which is as follows:

2 $$\varepsilon =\frac{\varDelta \bar{P}}{{E}_{{cell}}}{\left(\frac{{t}_{{creep}}}{{t}_{0}}\right)}^{\beta }$$

where $\Delta \bar{{\rm{P}}}=1/{{\rm{t}}}_{{\rm{creep}}}\int \triangle {\rm{p}}\left({\rm{t}}\right){\rm{dt}}$ represents the mean pressure difference in the microconstriction; ${{\rm{E}}}_{{\rm{cell}}}$ is the Young’s Modulus of cells; ${\rm{\beta }}$ is the power-law exponent, and the value is 0.1 ~ 0.5 of cell^22^. Since ${{\rm{t}}}_{0}$ is the timescale, which can be arbitrarily set to 1 s, Eq. (2) can be transformed into Eq. (3):

3 $${t}_{{creep}}={\left(\frac{{\varepsilon }_{e}{E}_{{cell}}}{\varDelta \bar{P}}\right)}^{\frac{1}{\beta }}$$

The cell area can be calculated as ${A}_{{cell}}=\pi \cdot {R}^{2}$. The deformation rate of cells can be rewritten as:

4 $${\varepsilon }_{e}=\frac{R-{R}_{e}}{R}=1-\frac{{R}_{e}}{R}=1-\frac{{R}_{e}\cdot \sqrt{\pi }}{\sqrt{{A}_{{cell}}}}$$

In constriction-based deformability cytometry, as shown in Eqs. (1)–(4), passage time is positively correlated with friction and negatively correlated with pressure and cellular deformability. Additionally, cell area has a positive relationship with cell passage time. The deformation index, which is defined as the degree of cell deformation within the microconstrictions and previously described in Eq. (5)^24^, was also utilized in our measurements:

5 $${Deformation}{{\_}}{index}=\left(H-W\right)/\left(H+W\right)$$

where H and W represent the length and width of a cell as it passes through a narrow microconstriction (Fig. 2b). The area of the cell body that is captured by a high-speed camera while the cell is within the microconstriction is referred to as the "area-in-constriction" (Fig. 2b).

To automate training set generation, multiobject tracking, segmentation, quantification of required parameters, and classification of cells with different metastatic potentials, we developed the ATMQcD computational framework (Fig. 2c). The ease of system operations enabled us to collect multivariate data on a single-cell resolution, such as passage time, cell size (area), area-in-constriction, and deformation index, using image processing.

### Generating training datasets and cell detection

**Fig. 3:**
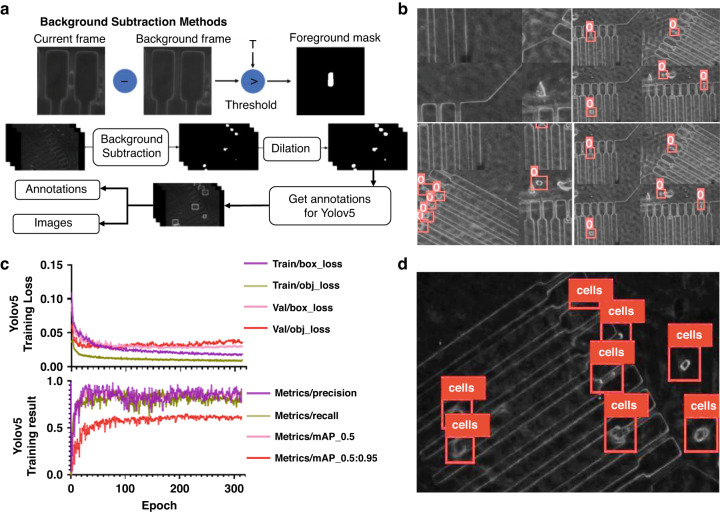
Training set generation and Yolov5-based model establishment. **a** The graphical principle of the background subtraction method (upper panel) and an overview of training dataset creation for Yolov5 (lower panel). **b** Representative images in the training set after being processed for mosaic augmentation. **c** The training loss, precision, recall, and mean average precision (mAP) of Yolov5. **d** Detection of cells by the established Yolov5 model

Generating training datasets and manually labeling them is typically a time-consuming process. Here, we developed an automated training set generation component for the ATMQcD computational framework to expedite the analytical process and enhance the transferability of the computational framework, which can reduce labeling time by up to 90%. As illustrated in Fig. 3a, background subtraction and thresholding can produce a clear foreground mask of cells, while dilation improves the quality of the cell boundary. To improve the model’s robustness, we applied Yolov5’s mosaic augmentation and other augmentations to the training set (Fig. 3b). The Yolov5-based model’s training loss, precision, recall, and mean average precision (mAP) are shown in Fig. 3c.

The Yolov5-based model demonstrated excellent performance in cell detection (Fig. 3d). Because high-speed cameras generate long time-lapsed videos with high frame rates, fast deep learning models are necessary, which the Yolov5-based model can effectively adapt to, significantly reducing detection time. The training parameters for Yolov5 were lr (learning rate) = 0.01; optimizer: SGD (stochastic gradient descent), momentum = 0.937; the training dataset comprised 160 images, and the validation dataset comprised 40 images. Yolov5 completed training for 314 epochs in 1.015 h.

During the practical operation of our system, we measured an average processing speed of 0.0113 s per frame, which is 1771.6% faster than the previously reported rate of another high-throughput algorithm for object detection called Faster R-CNN (0.2 s per frame)^25^. This result highlights the significant advantage of Yolov5 in cell detection.

### Cell tracking for quantification of deformability

**Fig. 4:**
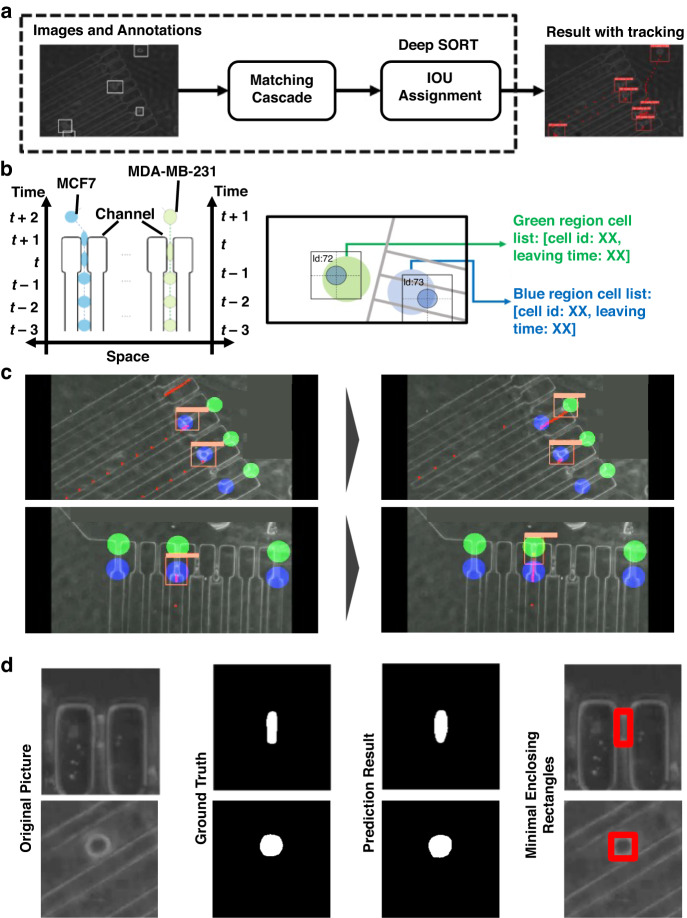
Cell tracking and quantification of cellular deformability measurements. **a** Brief demonstration of cell tracking based on Deep SORT. **b** Schematic representation of the application of green and blue detection regions for passage time quantification and how cells with different deformability could be differentiated by passage time. **c** Representative optical images demonstrating the running analysis program. The blue and green circles in these images represent the blue and green detection regions, respectively. Red dots behind each cell represent the tracks, while rectangular boxes mark the cell positions. **d** Segmentation of a cell from its original picture based on the trained ResUNet++ model

To establish a quantitative parameter for measuring cell stiffness, multiple parameters related to cell morphology and motility during deformation should be obtained and applied to fit the mechanical model based on power-law rheology^16,22^. Here, individual cells were identified and tracked using Yolov5-based object detection in combination with cell tracking. The Deep SORT algorithm was used for cell tracking based on the Yolov5 output (Fig. 4a)^26^.

To determine the passage time, focal regions were established in the entrance (blue region) and exit (green region) images (Fig. 4b, c). When a cell was detected in the blue region, the program recorded the cell’s identifier (ID) and entry time (Fig. 4b). The cell’s ID and the timestamp of detecting the cell in the green region were recorded as the time of exit (Fig. 4b, c). Each timestamp corresponds to a single video frame. Passage time was calculated by subtracting the first time the cell was recorded in the blue region from the last time it was recorded in the green region.

### Cell segmentation for morphological data

To quantify the deformation index, cell size, and area-in-constriction, we employed a deep learning model based on UNet to segment cells identified by YOLOv5. The program saved and processed images only when a cell was detected in the blue or green focus point to reduce processing time. After detecting a cell, the program obtained the cell’s minimal enclosing rectangles and the cell’s area (${A}_{{cell}}$) before entering the microconstrictions and the cell area inside the microconstrictions. The block unit in UNet is illustrated in Supplementary Fig. 5a.

We utilized the ResUNet++ segmentation model with a residual unit for identity mapping (Supplementary Fig. 5b) to achieve good segmentation results (Fig. 4d). The general architecture of our model is depicted in Supplementary Fig. 5c.

### Sensitive assessment of the metastatic potential of cancer cells using motility and morphometric measurements

We evaluated the microfluidic system’s ability to distinguish cells with different deformability by using two breast cancer cell lines, MCF7 and MDA-MB-231, with varying metastatic potential. Previous studies have shown that MDA-MB-231 cells are more invasive and deformable than MCF7 cells^7^. To compare the two cell lines, we determined the passage time, deformation index, and area in constriction.

**Fig. 5:**
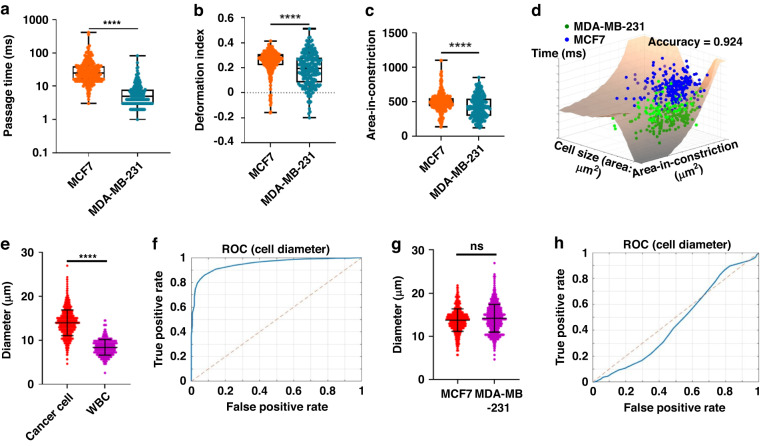
Sensitive assessment of the metastatic potential of cancer cells in heterogeneous blood samples using motility and morphometric measurements. **a** Box plot reflecting the difference in passage time for MCF7 and MDA-MB-231 cells. **b** Box plot reflecting the difference in the deformation index for MCF7 and MDA-MB-231 cells. **c** Box plot reflecting the difference in the area-in-constriction for MCF7 and MDA-MB-231 cells. **d** Three-dimensional plot showing the classifying efficiency of support vector machine (SVM) in classifying MCF7 and MDA-MB-231 using three-dimensional data (passage time, area-in-constriction, and original cell size) as input. The hyperplane dividing the two types of cells determined by SVM is displayed in three-dimensional space. Sample size in (**a**–**d**): n(MCF7) = 263; n(MDA-MB-231) = 281. **e** Box plot reflecting the difference in cancer cell diameter and white blood cells (WBCs). **f** The receiver operator characteristic (ROC) curve demonstrates the performance of classifying cancer and WBCs based on cell diameter. **g** Box plot reflecting the difference in cell diameter between MCF7 and MDA-MB-231 cells. **h** ROC curve demonstrating the performance of classifying MCF7 and MDA-MB-231 cells based on cell diameter. Statistical significance was calculated by unpaired two-tailed Student’s *t* test (*P* value > 0.05: ns; <0.05: *; <0.01: **; <0.001: ***; <0.0001: ****)

Our results demonstrate that MDA-MB-231 cells have a significantly shorter passage time than MCF7 cells, indicating that MDA-MB-231 cells have a higher metastatic potential and are better able to deform and pass through narrow spaces (Fig. 5a). The area under the receiver operating characteristic curve (AUC) for passage time was 0.937, indicating that it was an effective parameter for distinguishing between the two cell lines based on their deformability. In contrast, the deformation index and area-in-constriction were significantly higher in MCF7 (Fig. 5b, c). These parameters do not have strong predictive capabilities on their own, as evidenced by their lower AUC values (0.65 and 0.62, respectively) compared to passage time (Supplementary Fig. 6).

However, the overall prediction capabilities are expected to be robust when used as part of a multi-index classification approach in conjunction with other indices. To demonstrate this, we evaluated the accuracy of support vector machine (SVM) classification models based on various combinations of cellular deformability measurements. When considering both passage time and cell size, the SVM model achieved an accuracy of 0.89260 (Supplementary Table 3, Supplementary Fig. 7). However, incorporating additional parameters such as cell size and area-in-constriction into the model input improved the accuracy to 0.92394 (Supplementary Table 3, Fig. 5d). These findings demonstrate that high-dimensional data from motility and morphological measurements can enhance the classification of cells with varying metastatic potentials.

We further evaluated the potential of cell diameter gating to distinguish cancer cells from white blood cells in liquid blood biopsy samples. MCF7 and MDA-MB-231 cells were used to represent cancer cells, while white blood cells were separated from whole human blood by red blood cell lysis following procedures stated in previous literature^18^. We used the ATMQcD framework to determine an appropriate threshold for cell diameter. Our results demonstrated that our system achieved an accuracy of 89.5% in classifying cancer cells from white blood cells (Fig. 5e, f). Our findings suggest that cell diameter is an efficient parameter for accurately classifying cancer cells from white blood cells based on our deep learning algorithms. Further validation using tumor-derived cells or circulating tumor cells separated from patient blood is warranted to further confirm the efficiency of this diameter gating method in actual liquid biopsy samples.

We also tested the cell diameter between various metastatic subtypes (Fig. 5g, h) and demonstrated that MCF7 and MDA-MB-231 cells involved in this study had similar size distributions.

### Improving stratification of the cancer cell metastatic potential based on cell trajectories and multidimensional data

Previous studies utilizing cDC-based systems have mainly relied on passage time as the primary measure of deformability^10,27^. However, direct readouts such as passage time, deformation index, and area-in-constriction are affected by cell sizes, which limits their sensitivity in quantifying stiffness, especially in cells with heterogeneous sizes. Although some cDC-based systems have measured multiple parameters to improve sensitivity^28^, the multiparameter approach is more suited for cell-type classification rather than precise quantification of cell stiffness in clinical sample processing. Therefore, obtaining a deformability quantification independent of cell size is critical for the sensitive and effective evaluation of cancer cell invasiveness.

**Fig. 6:**
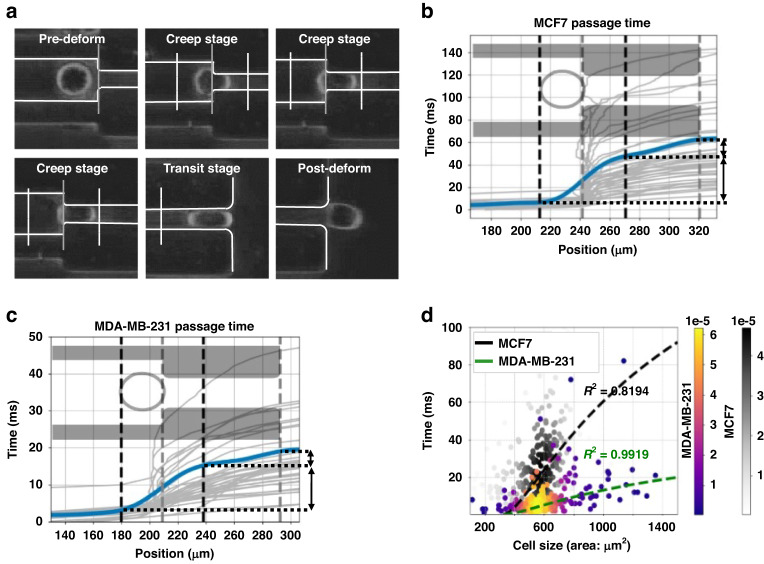
Trajectory analysis of breast cancer cells through the cDC+ATMQcD system. **a** Deformation of cells after entering the microconstriction. **b** Trajectory of MCF7 breast cancer cells in the position-time curve when traveling through the microconstriction. A schematic diagram of a microconstriction is shown in the line plot to demonstrate the corresponding relations between the position value on the x-axis and the actual position in the microconstriction. The range of the y-axis is 0–140 microseconds. The average time-position curves of MCF7 cells are displayed in blue. **c** Trajectory of breast cancer cells MDA-MB-231 in the form of the position-time curve when traveling through the microconstriction. The range of the y-axis is 0–50 microseconds. The average time-position curves of MCF7 cells are displayed in blue. The gray vertical dotted lines in (**b**, **c**) represent the entrance and exit of the microconstriction. **d** Plot of creeping time and cell size of MCF7 and MDA-MB-231 cells. Data points representing MCF7 and MDA-MB-231 cells are displayed in different colors. Colors of data points were also displayed according to the local density. Sample size: n(MCF7) = 263; n(MDA-MB-231) = 281. The theoretical size-t_creep_ curves calculated by Eq. (6) using the calculated c1 indices were plotted using dashed lines

Prior studies have reported group-based quantification of cell stiffness^16^. Here, we aimed to optimize the current quantification methods by implementing trajectory analysis and deep learning to measure the stiffness of single cells with greater sensitivity. We aimed to improve the sensitivity of evaluating cancer cell metastatic potential. To accomplish this, we recorded the detailed deformation process of each cell to obtain a more straightforward quantification of cellular deformability (Fig. 6a). We analyzed the cells’ trajectories in terms of their distance from the start of the creep phase region, and the time it took the cells to pass through this region, with the time-position curves of cells shown in Fig. 6b, c.

Using trajectory analysis, we determined the time required for cells to travel through the creep stage (tcreep). We applied the mechanical model of cells based on power-law rheology to calculate parameters directly proportional to the elastic modulus^16,22^, obtaining *t*_*creep*_. The specific value of the ${c}_{2}$ constant for our cDC device can be obtained as stated in ${c}_{2}={R}_{e}\,\cdot\, \sqrt{\pi }=10\mu m\,\cdot\, \sqrt{\pi }=17.7245$ μm (where 10 μm is the width of microconstriction). Based on c2, we quickly determined *c1* for the two cell types, which directly quantifies the stiffness of a group of cells, using Eq. (6) according to the collected data of *t*_*creep*_.

The power-law exponent β for cells typically falls between 0.1 and 0.5^16^. Here, we evaluated β ranging from 0.1 to 0.5 and applied Eq. (6) to assess their performance in actual fitting data of *t*_*creep*_ (Fig. 6d). We found that β = 0.5 resulted in the highest fitting performance in both cell types (Supplementary Fig. 8), with R-squared values equal to 0.9919 and 0.8194 for the actual fitting data of MDA-MB-231 and MCF7, respectively.

We calculated the theoretical *c1* indices for MCF7 and MDA-MB-231 cells using Eq. (6), with β equal to 0.5 and *c2* equal to 17.7245. Our results showed that the *c1* value for MCF7 cells was higher than that for MDA-MB-231 cells (Supplementary Table 4), indicating that MCF7 cells were stiffer than MDA-MB-231 cells and suggesting that MDA-MB-231 cells had a higher metastatic potential. These findings were consistent with these two cell types’ mechanical and physiological properties^7^.

We performed estimations of the elastic modulus for MCF7 and MDA-MB-231 cells to validate our system’s capabilities further. Through fluidic simulation on the cDC device, we determined that the average pressure drop (ΔP) across the microconstrictions was 162.82 Pa (Supplementary Fig. 9a). Utilizing this ΔP value and the c1 indices of the two cell types (Supplementary Table 4), we calculated the elastic modulus of MCF7 and MDA-MB-231 cells to be 2.881 kPa and 1.342 kPa, respectively (Supplementary Fig. 9b).

These values align closely with those reported in previous studies that employed single-beam acoustic tweezers (MCF7: 2.650 kPa; MDA-MB-231: 1.527 kPa) and atomic force microscopy (MCF7: 2.44 kPa; MDA-MB-231: 1.32 kPa)^29,30^. Furthermore, another study utilizing microfluidics with microconstrictions also obtained similar elastic modulus values for these two cell types (MCF7: approximately 2 kPa; MDA-MB-231: approximately 1.5 kPa) when using an inlet pressure of 100 Pa^8^. These consistent findings with the literature further validate the applicability and reliability of our system.

The performance of cDC technologies in assessing cell stiffness is influenced by the geometric dimensions of microconstrictions, making them more effective within specific size ranges. To determine the optimal size range for our system, we conducted linear regression analyses to examine the relationships between cell sizes and the elastic modulus of MCF7 and MDA-MB-231 cells (Supplementary Fig. 9c, d). We hypothesized that the dependent variable of the linear regression model (elastic modulus) for each cell type would fall within the mean elastic modulus ± the standard deviation (SD) when the independent variable (cell size) was within the optimal range.

Using this criterion, we determined the suitable size ranges for MCF7 and MDA-MB-231 cells (Supplementary Fig. 9c, d). We identified their overlap as the optimal size range for our system, which ranged from 547.1357 to 1034.096 μm². Correlation analysis demonstrated no significant correlation between the elastic modulus and cell sizes of MCF7 and MDA-MB-231 cells within this optimal size range (Supplementary Fig. 9e, f). The results indicated that applying the optimal size range for filtering cell data effectively eliminated the influence of cell sizes on the quantification of stiffness.

For clinical or industrial applications, the stiffness value derived from *t*_*creep*_ can directly assess the metastatic potential of each cell, and accompanying machine learning models built on high-dimensional profiling data of cell populations could provide a practical approach for patient stratification or staging.

### Maintaining robust evaluations in the presence of heterogeneous mixed samples

In clinical settings, tumors are typically heterogeneous tissues, meaning that cancer cells within the same tumor may exhibit different phenotypes, including varied metastatic potentials^31^. According to transcriptomic analysis, the proportion of invasive TNBC cells in highly metastatic triple-negative breast cancer (TNBC) tumors is usually greater than 20%, with some cancer cell populations reaching up to 80%^32^.

To showcase the capacity of our system to detect phenotypic heterogeneity among cancer cells in real clinical samples, we simulated the compositions of invasive cancer cells in triple-negative breast cancer (TNBC) by creating four groups of mixed samples. These groups, labeled A, B, C, and D, represent different MCF7:MDA-MB-231 concentration ratios of 1:4, 4:1, 1:9, and 9:1, respectively. Cells were found to exhibit a similar size distribution, within the optimal range of 547.1357–1034.096 μm² (Supplementary Fig. 10a). It is important to note that the size of cells does not correlate with the c1 index (Supplementary Table 5).

**Fig. 7:**
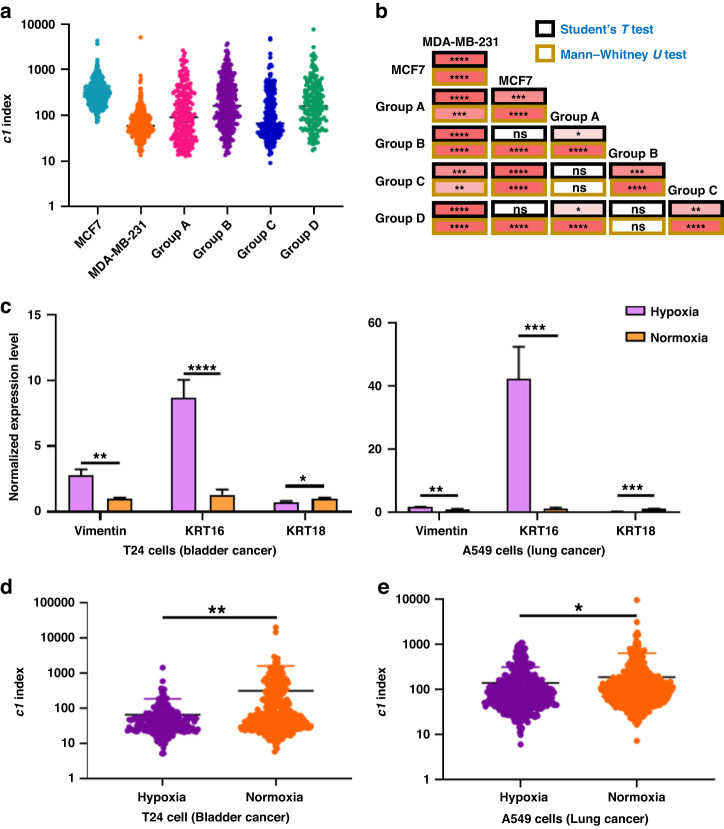
Quantification of cellular deformability for distinguishing cells with different metastatic potentials. **a** Comparing *c1* indices of MCF7, MDA-MB-231, and four groups of mixed samples. The ratio of Group A was MCF7: MDA-MB-231 = 1:4, Group B was MCF7: MDA-MB-231 = 4:1, Group C was MCF7: MDA-MB-231 = 1:9, and Group D was MCF7: MDA-MB-231 = 9:1. Axes showing data of the *c1* index are displayed with a log10 scale. Sample size: n(MCF7) = 316; n(MDA-MB-231) = 310; n(Group A) = 215; n(Group B) = 310; n(Group C) = 248; n(Group D) = 175. **b** Heatmap demonstrates the groupwise difference’s statistical significance in the *c1* index. The statistical significance was calculated by Student’s t-test (marked in black rectangles) and Mann‒Whitney U test (marked in deep-yellow rectangles). **c** Normalized expression levels of vimentin, *KRT16*, and *KRT18* in hypoxia- and normoxia-treated T24 and A549 cell lines. **d** Comparing *c1* indices of normoxia- and hypoxia-treated bladder cancer cells (T24) based on Student’s *t* test. Sample size: n(hypoxia-treated T24) = 196; n(normoxia-treated T24) = 392. **e** Comparing *c1* indices of normoxia- and hypoxia-treated lung cancer cells (A549) based on Student’s t-test. Sample size: n(hypoxia-treated A549) = 405; n(normoxia-treated A549) = 605. (*P* value > 0.05: ns; 0.05: *; <0.01: **; <0.001: ***; <0.0001: ****)

The results presented in Fig. 7a indicate that as the proportion of metastatic cancer cells increased, the c1 index of the cell population decreased. The average c1 index values were as follows: 390.5 for MCF7 only, 377.4 for Group D, 369.7 for Group B, 257.7 for Group A, 216.3 for Group C, and 99.0 for MDA-MB-231 only. This demonstrates that the c1 index effectively reflects the proportion of highly metastatic cancer cells (MDA-MB-231) in cellular samples.

To assess the significant differences in the c1 index among the six groups of samples, we conducted both Student’s t-test and Mann‒Whitney U test in a groupwise manner. Figure 7b illustrates the results of these tests. According to the Mann‒Whitney U test, when the proportion of highly metastatic MDA-MB-231 cells ranged from 10% (Group D) to 20% (Group B) of the cell population, the c1 index was significantly lower than that of pure MCF7 cells. When the proportion of metastatic MDA-MB-231 cells increased to 80% (Group A) and 90% (Group C), the Mann‒Whitney U test and Student’s *t* test identified significant differences.

These findings serve as clear evidence of the potential of our system in identifying phenotypic heterogeneity and in practical applications^33^, such as patient stratification or staging based on metastatic potential.

It is important to highlight that MCF-7 and MDA-MB-231 cell lines are commonly used as standard models due to their well-established differences in metastatic potential. To evaluate the utility of our systems in clinical applications, we anticipate further validation involving patient-derived cancer cells.

### Broad applicability of the integrated system demonstrated with lung and bladder cancer cell samples

Having confirmed the high efficiency of our cDC + ATMQcD system in breast cancer, we extended our evaluation to other cancer types, namely, lung (A549) and bladder (T24) cancer, to assess the system’s broad applicability. We enhanced the metastatic potential of each cell line by maintaining them under hypoxic conditions, which has been shown to induce epithelial-mesenchymal transition (EMT) and cancer cell invasiveness^34,35^.

We employed real-time quantitative reverse transcription polymerase chain reaction (qRT‒PCR) to assess the expression levels of three biomarkers, *vimentin*, *KRT16*, and *KRT18*, associated with metastasis and EMT in cancer cells following a 48-h hypoxia treatment. Previous studies have identified vimentin and KRT16 as significant factors associated with metastasis and EMT^36–39^, whereas KRT18 has been shown to correlate negatively with EMT^40–42^. Our results confirmed that hypoxia treatment upregulated the expression of *vimentin* and *KRT16* while downregulating the expression of *KRT18* in both cancer cell types (Fig. 7c), indicating the upregulation of EMT and metastasis.

After confirming the upregulation of biomarkers reflecting EMT and metastasis following hypoxia treatment, we assessed both hypoxia-treated and normoxia-treated cancer cells within a similar size range using our cDC + ATMQcD system (Supplementary Fig. 10b, c). We evaluated the suitability of the power-law exponent β = 0.05 for A549 and T24 cells by varying β from 0.1 to 0.5. Using Eq. (6), we assessed the performance of different β values in fitting the t_creep_ data. Supplementary Fig. 11 demonstrates that when β = 0.5, the R-squared values for fitting the A549 and T24 data were 0.9423 and 0.9923, respectively. This indicates that a value of β = 0.5 is appropriate for accurately quantifying the stiffness of both A549 and T24 cells. According to Fig. 7d, e, hypoxia treatment significantly decreased the c1 index of lung and bladder cancer cells, indicating decreased cellular stiffness. Correlation analysis revealed no significant correlation between the sizes of these cells and their corresponding *c1* index values (Supplementary Table 5). The observed decrease in the *c1* index provides robust evidence to confirm the ability of our cDC + ATMQcD system to evaluate the metastatic potential of multiple cancer types.

Overall, our cDC + ATMQcD system provides a robust quantification of cellular stiffness using the *c1* index, which is sensitive enough to detect changes in cell invasiveness caused by phenotypic differences or microenvironmental changes across multiple cancers.

## Discussion

Cell deformability is a key feature associated with many diseases, including cancer, cardiovascular disease, and malaria^3–8,43^. Numerous techniques have been developed to measure cellular deformability, including atomic force microscopy, microaspiration, optical stretching, and parallel-plate rheology (Supplementary Table 1). However, these approaches have limited throughput, typically measuring only 10 cells per minute, and require expensive, complex, and bulky equipment^44^. Therefore, the challenges in accurately measuring cellular deformability continue to hinder their translation for clinical and industrial applications.

In recent years, microfluidic deformability cytometry has emerged as the most promising approach to measuring cellular deformability due to the introduction of microfluidic technologies (Supplementary Table 1) that allow for extremely high throughput. However, the future of microfluidics-based deformability cytometry lies in developing sensitive, low-cost, high-throughput, user-friendly, and mass-producible systems for applications outside of research laboratories. Achieving a balance across these criteria can be challenging since they are often mutually exclusive. The xDC and sDC systems are the most high-throughput models for measuring cellular deformability; however, they rely on fluid flow forces to induce cell deformation. These techniques require strong fluidic forces to deform the cells effectively.

Consequently, in the xDC and sDC methods, the cells experience rapid movement across the microchannels, resulting in higher velocities. This necessitates using high-frame-rate cameras to capture rapid cell motion passage accurately. In contrast, the cDC approach involves a slower movement speed of objects due to its reliance on direct contact between cells and the channel walls.

The design of cDC devices intentionally incorporates narrow channels that exert pressure on cells, causing them to deform and pass through the channels gradually. This fundamental distinction in operating principles is also evident in previous studies. For example, investigations involving sDC and xDC typically employ high-speed cameras capable of operating at frame rates as high as 10,000 frames per second^14,15^, which can result in increased costs, especially in resource-limited settings or developing countries. On the other hand, cDC can be adequately captured using cameras with lower frame rates of fewer than 1000 frames per second^16,45^. However, it is important to note that the throughput of cDC is comparatively lower than that of xDC and sDC methods^16^.

To overcome these challenges, we developed a cellular deformability measuring system that balances these critical criteria for field applications. Our ATMQcD computational framework allows for high-throughput and automatic sample processing and data analysis while remaining low-cost and straightforward to fabricate. By combining cutting-edge technologies for object detection (Yolov5), tracking (Deep SORT), and segmentation (ResUNet++), the ATMQcD computational framework can capture multiple fast-moving objects in one field of view and enable high-throughput cell measurement. ResUNet++ can be replaced with other segmentation deep learning models in future applications if updated versions are developed. With the help of ATMOcD, we obtained multiple parameters related to cell morphology and motility, including passage time, cell size, and area-in-constriction. We developed a simple SVM classifier based on these parameters, which achieved a high accuracy of 0.934 in breast cancer cells with different metastatic potentials. Further studies can incorporate more parameters with SVM for analysis. Furthermore, the ATMQcD framework can be combined with biomechanical analysis based on cell trajectories to quantify cell stiffness (*c1* index) proportional to the cell elastic modulus.

The cDC + ATMQcD system is a groundbreaking platform that integrates a deep-learning computational framework in quantifying single-cell deformability. By combining microfluidics and deep learning, the system achieved a high throughput of approximately 25,000 cells per minute, allowing for high sensitivity, low cost, and ease of operation. In the future, the cDC + ATMQcD system is expected to find wide applications, including liquid biopsy, drug testing, and investigation of disease mechanisms, by integrating with microfluidics for biomarker enrichment^46,47^ and in-vitro disease models^48,49^.

The system was validated across various cancer types, such as breast, lung, and bladder cancers, demonstrating its potential in evaluating cancer cells’ metastatic potential based on stiffness after treatment. This broad applicability highlights the clinical relevance of the cDC + ATMQcD system. Furthermore, the system addresses previous deformability cytometric system limitations in processing throughput and cost without compromising sensitivity. Clinical trials are in progress to validate the cDC + ATMQcD system’s potential as a promising tool for routine clinical and research applications.

## Materials and methods

### Design and fabrication of cellular deformability probing chip

The pattern of microchannels was drawn by AutoCAD. Fluid flow was simulated by COMSOL Multiphysics 5.5 using the laminar flow module. An extremely fine mesh was built for the simulation to obtain precise results. During the simulation, polydimethylsiloxane (PDMS) was applied as the material for the channel walls. The microchannel layer was developed using standard photolithography and soft lithography with a negative photoresist (Cat#SU-8 2025, MicroChem, United States) and PDMS (Cat#01673921, Dow, United States), following the instructions as indicated and as previously reported^18,19^. When fabricating the master mold, the photoresist was spin-coated on a silicon wafer with a spinner (Cat# KW-4A, SETCAS Electronics, China) by ramping to 500 rpm for 5–10 s with a 100 rpm/s acceleration and then ramping to 2500 rpm with a 300 rpm/s acceleration for 30 s, resulting in a 30 μm-thick photoresist layer.

The master mold was surface-modified by silanization using trichloro (1H, 1H, 2H, 2H-perfluorooctyl) silane (Cat#448931-10G, Sigma‒Aldrich, Germany) before soft lithography^50^. During soft lithography, PDMS was poured onto the silanized silicon wafer, followed by stringent degassing using a vacuum pump (Cat#167300-22, Rocker, China). The inlet and outlet of the microfluidics were punched on the PDMS microchannel layer before bonding to a glass cover slide.

### Cell culture and on-chip processing

The lung cancer cell line A549, bladder cancer cell line T24, and two breast cancer cell lines, MCF-7 and MDA-MB-231 (ATCC, United States) were used in this study. MCF-7 originated from estrogen and progesterone receptor-positive breast cancer subtypes, while MDA-MB-231 originated from a more aggressive triple-negative subtype^51^. All the cell lines were adherent cells cultured in high-glucose Dulbecco’s Modified Eagle Medium (DMEM) (Cat#10566-016, Thermo Fisher, United States) with 10% fetal bovine serum (FBS) (Cat#10270106, Thermo Fisher, United States) in a 37 °C incubator (5% CO2). Hypoxia treatment of lung and bladder cancer cell lines was performed by incubating the cells in a hypoxia incubator (Cat#381, Thermo Fisher, United States) under 2% O2 at 37 °C.

Before processing with microfluidic chips, cells were detached by trypsin (Cat#R001100, Thermo Fisher, United States) and diluted to approximately 5 × 10^5^ cells/mL in DMEM culture media with 2.5% (v/v) bovine serum albumin (Cat#B14, Thermo Fisher, United State). Before each run, the microfluidic chip was washed with 2.5% bovine serum albumin under a flow rate of 20 μL/min for 10 min, followed by washing with phosphate-buffered saline at 50 μL/min for 2 min. Air bubbles were removed from microchannels before priming cells into the chip. After washing, the cells were primed into the microfluidics at a 50 μL/min flow rate. A representative timelapse showing the optimal flow conditions in the cDC device is shown in Supplementary Video 1. A portable and low-cost high-speed camera (MV-A5031MU815, Dahua Technology, China) operated at 988 frame-per-second (fps) was mounted to the microscope to record images for analysis.

The fluidic simulations revealed that one-third of the microconstriction-related device fouling in a single cDC device increased the flow velocity by approximately 4 μm/s and the fluidic pressure at microconstrictions by 20 Pa (Supplementary Fig. 12a). To investigate the impact of microconstriction-related device fouling on cell measurements, we compared the c1 indices of cells from the same batch using the same cDC device under different fouling statuses. Specifically, we recorded images with 1, 2, 3, or 4 fouling within the same group of microconstrictions and found no significant difference in the measurements (Supplementary Fig. 12b). To ensure sensitivity, if over 3 microconstrictions in one group had microconstriction-related device fouling, then the recorded images were discarded.

### Design of the ATMQcD platform

The major measurements for evaluating cellular deformability include passage time and deformation index. The definition of these two measurements is shown in Fig. 2a, b. To achieve automated detection and quantification of these measurements, we developed a computational framework based on deep learning called ATMQcD (Fig. 2c).

The major elements in this framework include (i) the background subtraction method to obtain each cell’s position; (ii) training of Yolov5^52^ using images and annotation from the background subtraction method; (iii) object tracking based on conjugating Yolov5 and deep SORT^26^; (iv) calculation of the passage time using Yolov5 and deep SORT; (v) manual setting of the focus point; (vi) cutting cell images (size of pixel: $200\times 200$ or $50\times 50$) when the cells were crossing the focus points; (vii) training of ResUnet++ using images obtained in step (vi); (vii) calculating deformation index and size of the cell by ResUnet++^53^; and (viii) connecting the passage time and the area size of each cell.

In this framework, Yolov5 was used to detect the cell and track the trajectory of the cell by Deep SORT. After recording each cell’s position and trajectory, ResUNet++ was applied to calculate each cell’s area. The background subtraction process was implemented by the OpenCV module of Python 3.8. Yolov5 was implemented by PyTorch. ResUNet++ was implemented by PyTorch. Deep SORT was implemented by PyTorch.

### Generating training datasets and cell detection

Establishing a deep-learning-driven automated system requires a large amount of input as the training set. However, manual annotation of cells is labor-intensive and does not fit the frequently changed imaging settings in different field applications. In light of this problem, our ATMQcD platform applied background subtraction, which could detect moving objects from the difference between the current frame and reference frame^54^, to efficiently generate a large amount of training data. Background-subtracted images were transformed into binary images by thresholding, followed by dilation to increase the boundary of the regions of foreground pixels and reduce noise.

### Classifier based on SVM

A support vector machine (SVM) is a supervised machine learning model that uses classification algorithms for two-group classification problems. After giving SVM model sets of labeled training data for each parameter, the algorithm can categorize different cell types. In this article, the kernel function uses ‘poly’, degree = 3, coef0 = 1, and C = 5.

### Trajectory and deformation analysis of cells

We used the ATMQcD computational framework to perform trajectory and deformation analysis on the cells to assess their stiffness accurately. In this study, by collecting data from 526 MCF7 cells and 562 MDA-MB-231 cells, we found that the average diameters of MCF7 and MDA-MB-231 were 12.75 and 13.15 μm, respectively. Therefore, we defined the region of the creep stage as 12.95 μm before and after the entrance of a microconstriction. In this way, we can quantify the *t*_*creep*_ for each cell involved.

As stated, we have obtained the relationship between *t*_*creep*_, Young’s Modulus (*E*_*cell*_), and the parameter. ${\varepsilon }_{e}$ in Eq. (3):

3 $${t}_{{creep}}={\left(\frac{{\varepsilon }_{e}{E}_{{cell}}}{\varDelta \bar{P}}\right)}^{\frac{1}{\beta }}$$

The parameter ${\varepsilon }_{e}$ was defined by Eq. (4) as follows:

4 $${\varepsilon }_{e}=\frac{R-{R}_{e}}{R}=1-\frac{{R}_{e}}{R}=1-\frac{{R}_{e}\,\cdot\, \sqrt{\pi }}{\sqrt{{A}_{{cell}}}}$$

where constant ${c}_{1}={{E}_{{cell}}}^{\frac{1}{\beta }}/{\varDelta \bar{P}}^{\frac{1}{\beta }}$, and ${c}_{2}={R}_{e}\,\cdot\, \sqrt{\pi }$. $\varDelta \bar{P}$. The mean pressure drop across the microconstriction is assumed to be a constant in each experimental run under the same infusion flow rate. Therefore, *c1* can be seen as a measurement proportional to the cell elasticity, while *c2* is a constant proportional to the width of the microconstriction. The power-law exponent $\beta$ equals 0 when the material is purely elastic and equals 1 when the material is a purely viscous fluid. In cells, $\beta$ usually falls into 0.1–0.5^16^.

Set y = *t*_*creep*_, the equation for cell area and creeping time can be written as:

6 $$y=f\left({A}_{{cell}}\right)={t}_{{creep}}={c}_{1}\,\cdot\, {\left(1-\frac{{c}_{2}}{{{A}_{{cell}}}^{\frac{1}{2}}}\right)}^{\frac{1}{\beta }}$$

$f\left({A}_{{cell}}\right)$ means that the independent variable of the function is ${A}_{{cell}}$.

### qRT‒PCR for biomarkers of EMT and metastasis

RNA extraction, reverse transcription, and qPCR were conducted based on the RNA extraction kit (Cat# R401-01), HiScript III All-in-on RT SuperMix (Cat# R333-01), and ChamQ Universal SYBR qPCR kit (Cat# Q711-02) from Vazyme, China, respectively. One microgram of RNA was used for each reverse transcription reaction. The qPCR reactions were conducted by a BIO-RAD CFX Real-Time PCR machine. The baseline threshold, automatically calculated by Bio-Rad CFX Manager (Version 3.1), was adopted to retrieve the quantification cycle (Cq) values. *GAPDH* was used as the reference gene for normalization. Primers *for GAPDH*, vimentin, *KRT16*, and *KRT18* are shown in Supplementary Table 5.

### Supplementary information

Supplementary Materials

Supplementary Vedio 1

## Data Availability

The original contributions presented in the study are included in the article/Supplementary Material. Further inquiries can be directed to the corresponding author.
