# Supplementary material for: A deformability-based biochip for precise label-free stratification of metastatic subtypes using deep learning
Source: Microsyst Nanoeng. 2023 Sep 28;9:120. doi: 10.1038/s41378-023-00577-1 (PMC10539402; doi:10.1038/s41378-023-00577-1)
Supplement: Supplementary file 1 — Supplementary Materials [file 41378_2023_577_MOESM1_ESM.docx]

**Supplementary Materials**

**Supplementary Tables**

**Supplementary** **Table 1: Comparison of current systems for deformability cytometry.** AFM: Atomic force microscopy; cDC (constriction-based deformability cytometry); sDC (shear flow deformability cytometry); xDC (extensional flow deformability cytometry); ATMQcD: automatic training set generation, multiple object tracking, segmentation, and cellular deformability quantification

| **Type** | **System category** | **Measuring location** | **Throughput** | **Cost** | **Accuracy** | **Ref** |
| --- | --- | --- | --- | --- | --- | --- |
| Conventional method | **AFM** | At the cell surface | 1~20 cells per hour | High | Not sensitive | ^45^ |
| Conventional method | **Optical stretching** | Whole suspended cell | 60–300 cells per hour | High | Not sensitive | ^46^ |
| Conventional method | **Micro-aspiration** | Whole suspended cell | 20 cells per hour | Low | Sensitive | ^47^ |
| Microfluidics-based method | **xDC** | Whole suspended cell | > 1,000 cells per second | High | Not sensitive | ^14^ |
| Microfluidics-based method | **sDC** | Whole suspended cell | > 100 cells per second | Middle | Not sensitive | ^48^ |
| Microfluidics-based method | **cDC** | Whole suspended cell | > 1,000 cells per hour | Low | Sensitive | ^23^ |
| Microfluidics-based method | **Parallelized cDC** | Whole suspended cell | 6,000-10,000 cells per hour | Low | Sensitive | ^16,24,49^ |
| Microfluidics-based method | **cDC + ATMQcD system** | Whole suspended cell | ~ 25,000 cells per minute | Low | Sensitive | **Our work** |

**Supplementary** **Table 2: Comparison of current methods for measuring cancer cells’ metastatic potential.** qRT-PCR: quantitative reverse transcriptional polymerase chain reaction.

| **Method** | **Biomarker** | **Time consumption** | **Capable of evaluating cancer cell compositions** | **System cost** | **Ref** |
| --- | --- | --- | --- | --- | --- |
| Transwell assays | Cell migration | > 12 hours | No | Low (including microscope) | ^1^ |
| qRT-PCR | Gene-based | 6 to 8 hours | No | Medium (including PCR machines and reagents) |  |
| Single-cell RNA sequencing | Gene-based | > 1 day | Yes | Very High | ^2,3^ |
| cDC+ATMQcD | Cellular deformability | < 3 hours | Yes | Low ( including microscope and high-speed camera that costs less than 400 USD) | Our system |

**Supplementary** **Table 3: Accuracies of support vector machine models built on different combinations of input measurements in classifying MCF7 and MDA-MB-231.**

| **Input measurements** | **Accuracy** |
| --- | --- |
| Passage time | 0.87407 |
| Cell size | 0.54260 |
| Area-in-constriction | 0.63758 |
| Deformation index | 0.67114 |
| Passage time + Deformation index | 0.89709 |
| Passage time + Cell size | 0.89260 |
| Passage time + Cell size + Deformation index | 0.91275 |
| Passage time + Cell size + Area-in-constriction | 0.92394 |

**Supplementary** **Table 4: The value of c1 and c2 fitting by MATLAB.** 95% confidence interval of *c1* indexes were shown in the brackets.

| **Cell type** | **c1** | **c2** |
| --- | --- | --- |
| MCF7 | 312.7 (288.1, 337.3) | 17.725$\mu m$ |
| MDA-MB-231 | 67.85 (62.37, 73.33) | 17.725$\mu m$ |

**Supplementary Table 5 Correlations between cell sizes.** Data were obtained from cells within the optimal size range (547.1357 μm^2^ to 1034.096 μm^2^) and c1 index.

| Sample Group | Pearson Correlation Coefficient | Coefficient of Determination (R-squared) |
| --- | --- | --- |
| MCF7 | -0.0821 | 0.00674 |
| MCA-MB-231 | 0.1134 | 0.01287 |
| Mixed Group A | -0.1996 | 0.03983 |
| Mixed Group B | -0.0745 | 0.005549 |
| Mixed Group C | -0.0937 | 0.008779 |
| Mixed Group D | 0.0170 | 0.0002905 |
| A549 Hypoxia | 0.0348 | 0.001214 |
| A549 Normoxia | 0.0854 | 0.007287 |
| T24 Hypoxia | -0.0162 | 0.0002613 |
| T24 Normoxia | -0.0179 | 0.0003194 |

**Supplementary Table 5: Primers for qRT-PCR.**

| **Gene** | **Forward / Reverse** | **Sequence (5’ to 3’)** |
| --- | --- | --- |
| *GADPH* | Forward | GTCTCCTCTGACTTCAACAGCG |
|  | Reverse | ACCACCCTGTTGCTGTAGCCAA |
| Vimentin (*VIM*) | Forward | AGGCAAAGCAGGAGTCCACTGA |
|  | Reverse | ATCTGGCGTTCCAGGGACTCAT |
| *KRT16* | Forward | GACCGGCGGAGATGTGAAC |
|  | Reverse | CTGCTCGTACTGGTCACGC |
| *KRT18* | Forward | GCTCAGATCTTCGCAAATACTGT |
|  | Reverse | CTTCCTCTTCGTGGTTCTTCTTC |

**Supplementary Figures**


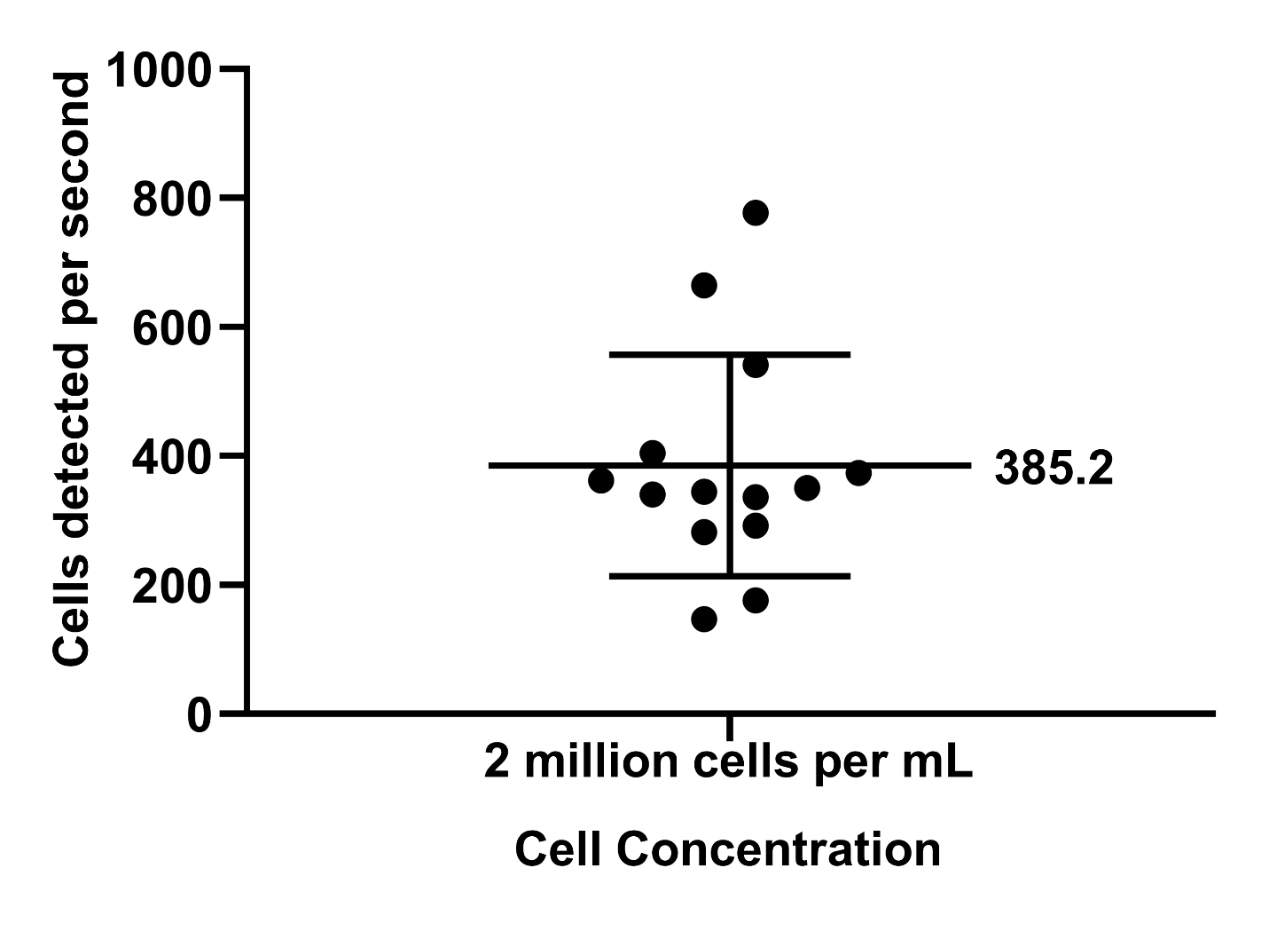


**Supplementary Figure 1 Determination of maximal throughput for the constriction-based deformability cytometry (cDC) device.** Each dot demonstrates the average cells detected per second for one video (8 to 17 seconds). Numbers demonstrate mean values of cells-detected-per-second obtained from samples of the two concentrations. Bladder cancer cell line T24 was applied for this test.


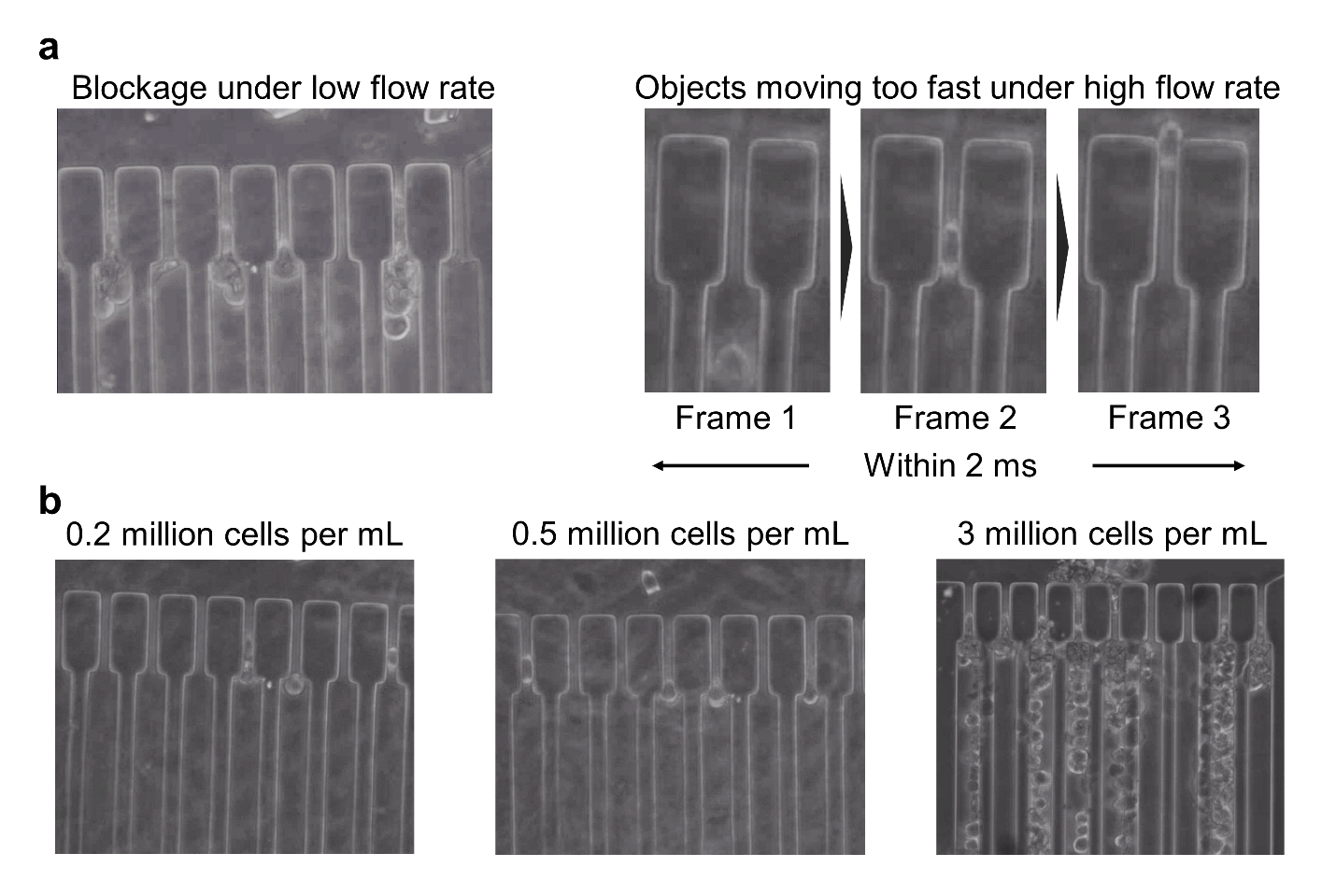


**Supplementary** **Figure 2 Optimization of flow rate and cell concentration. a** Device running under 30 μL/min (left) and 70 μL/min (right). **b** Device running with different concentrations of cells. Under high concentrations of cells, cells would quickly form aggregations in microconstrictions, leading to severe blockage. On the other hand, if the concentration were too low, there would be many empty microconstrictions, leading to low throughput.


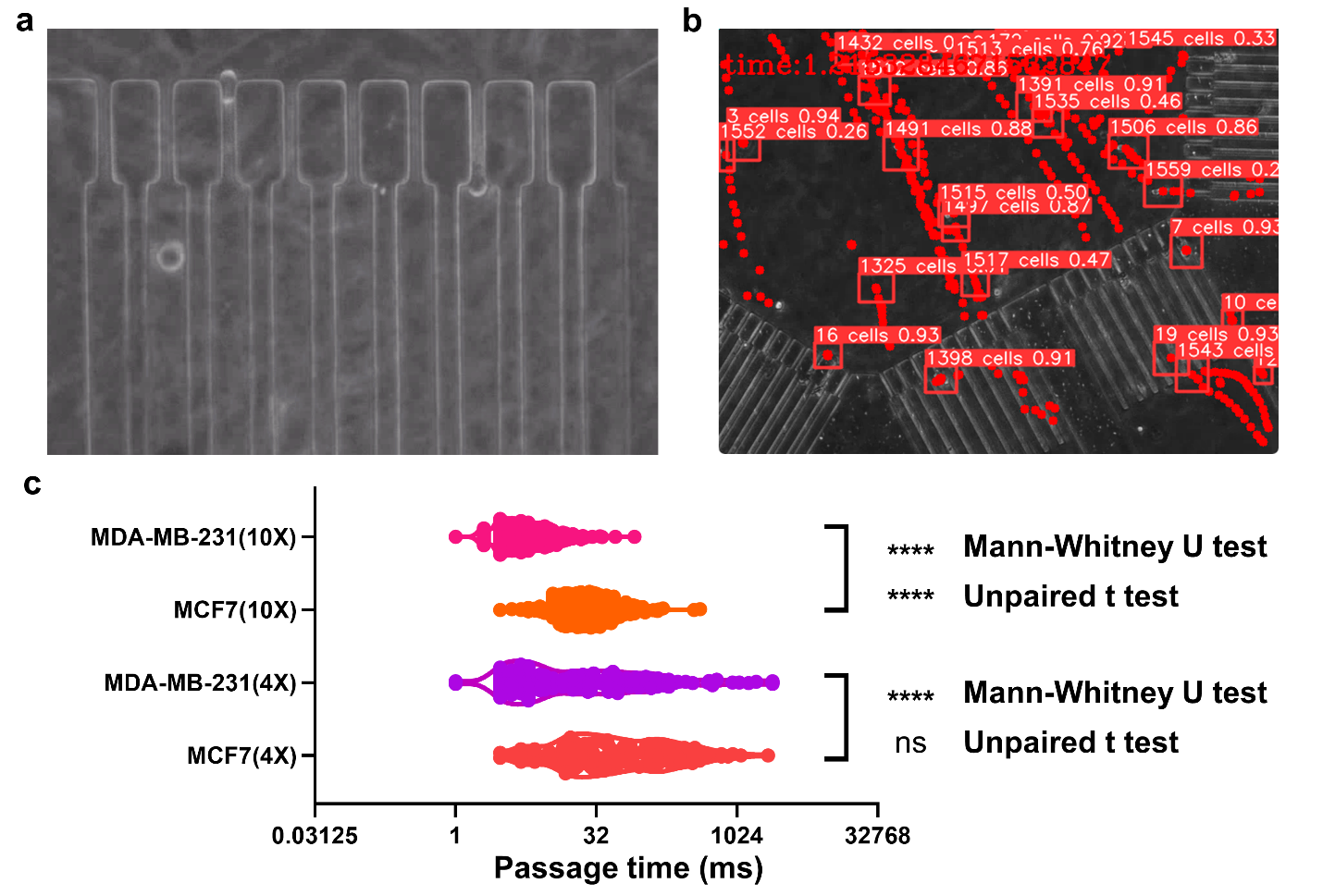


**Supplementary Figure 3 Performance of the cDC+ATMQcD system under various imaging parameters. (a)** A representative image of 9 microconstrictions in a single field under a 10X objective lens. **(b)** A representative image of 36 microconstrictions in a single field under a 4X objective lens, with cells (marked in red rectangles) and their moving tracks (marked by red dots) detected by Yolov5 and Deep SORT. **(c)** Comparison of MCF7 and MDA-MB-231 cell passage time based on images taken by a 10X and 4X objective lens, respectively. Statistical significance was determined by unpaired two-tailed Student’s t-test and Mann-Whitney U test (P-value >0.05: ns; < 0.05: *; <0.01: **; <0.001: ***; <0.0001: ****).

**
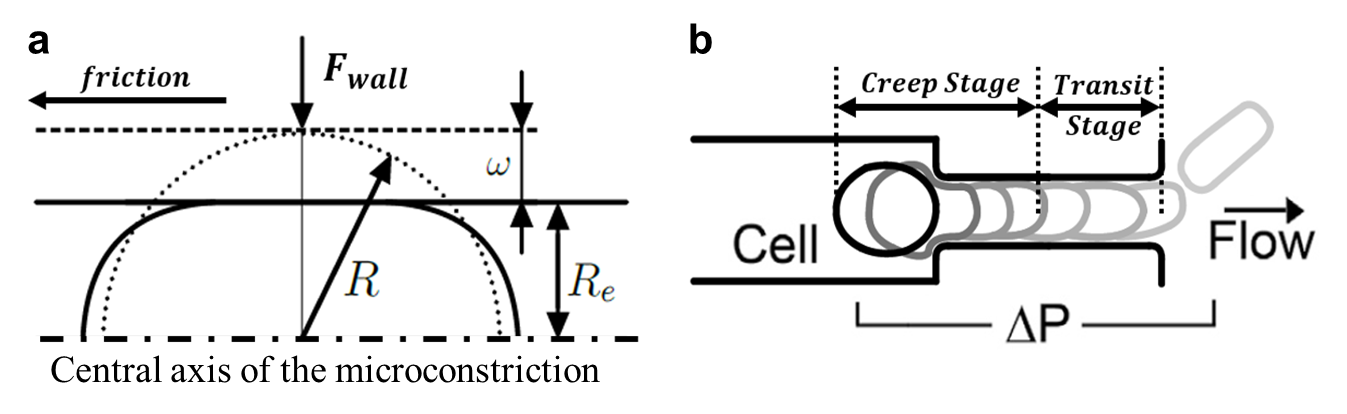
 Supplementary** **Figure 4 The deformation analysis of cells. (a)** The structure of cells after full entry into microconstriction. **(b)** The structure of the microconstriction and the stages of creep and transit stage of cells when the cell passes through the microconstriction.


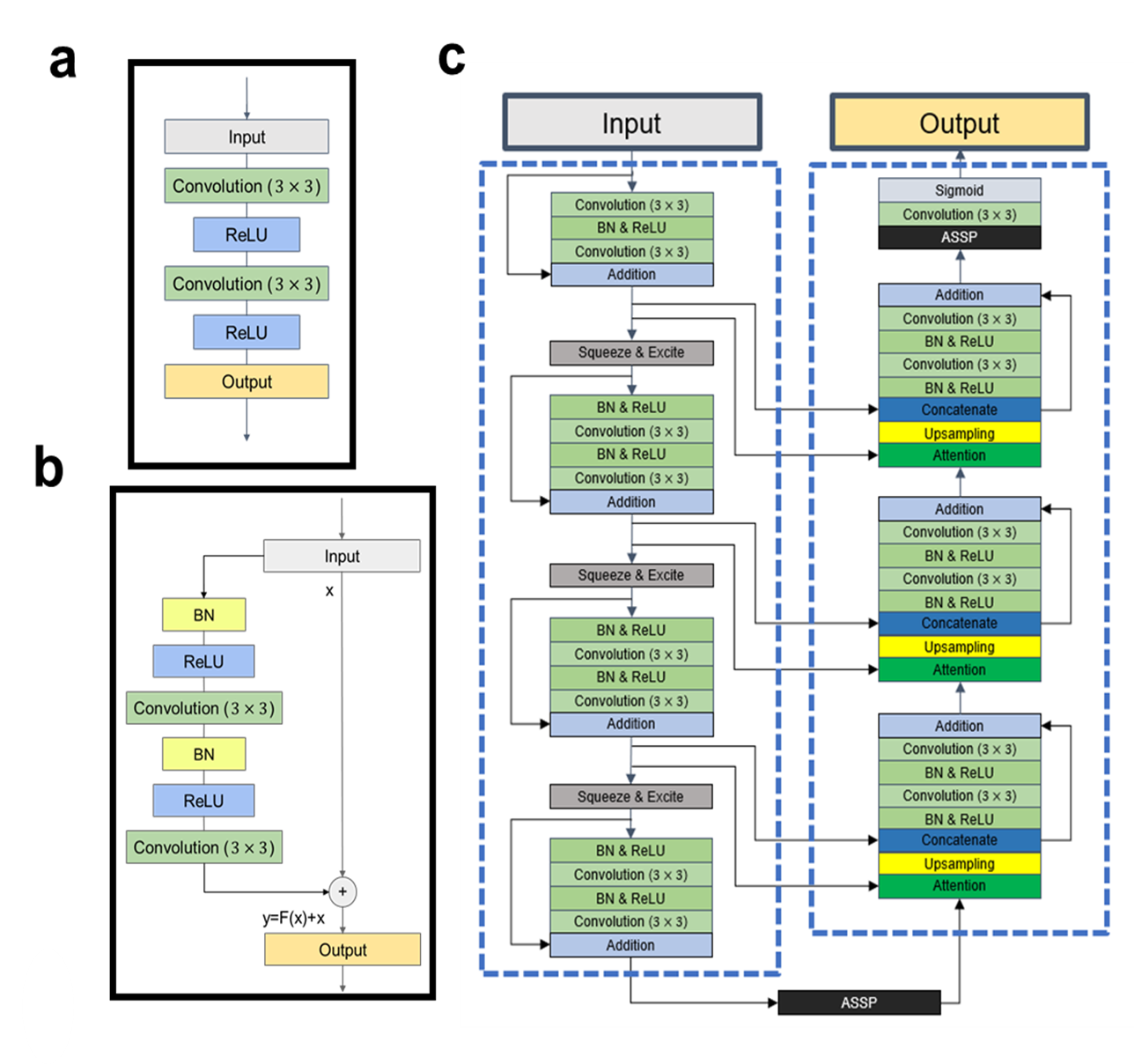


**Supplementary** **Figure 5 The architecture of ResUnet ++. (a)** Blocks unit used in Unet. **(b)** Residual unit with identity mapping used in ResUnet. **(c)** The architecture of ResUnet ++.


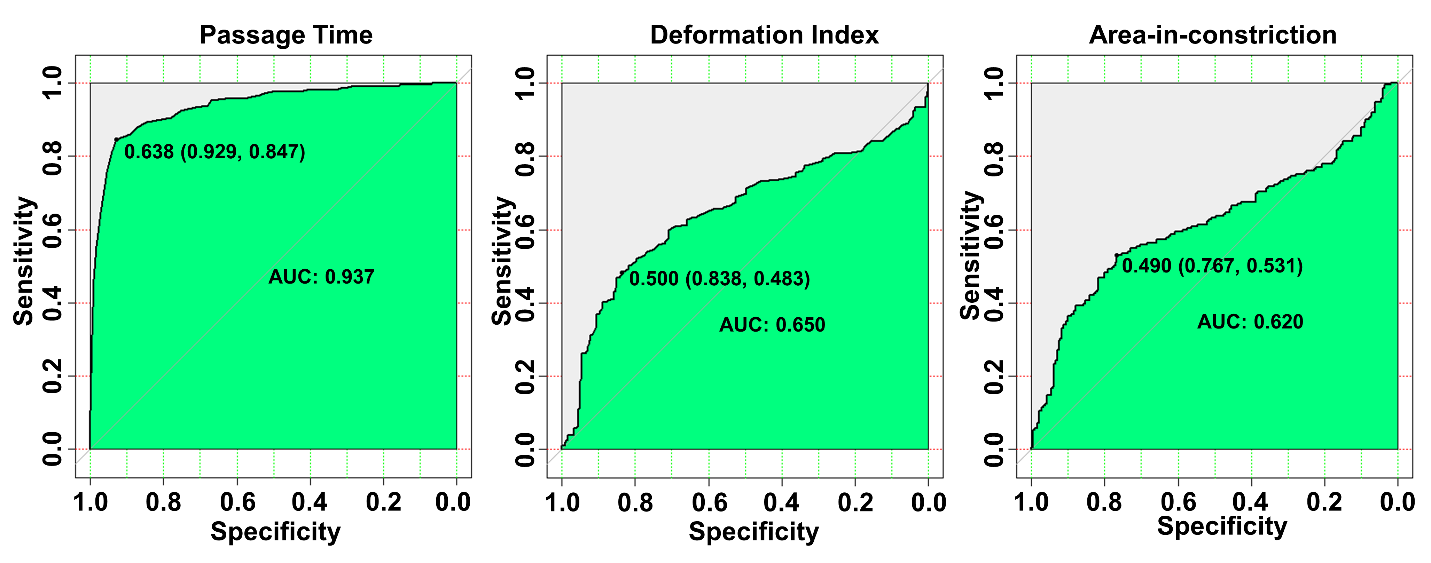


**Supplementary** **Figure 6 Receiver operator characteristic (ROC) curves of deformability-related parameters.** The area under ROC curves (AUC) of respective parameters was displayed in each corresponding subplot.


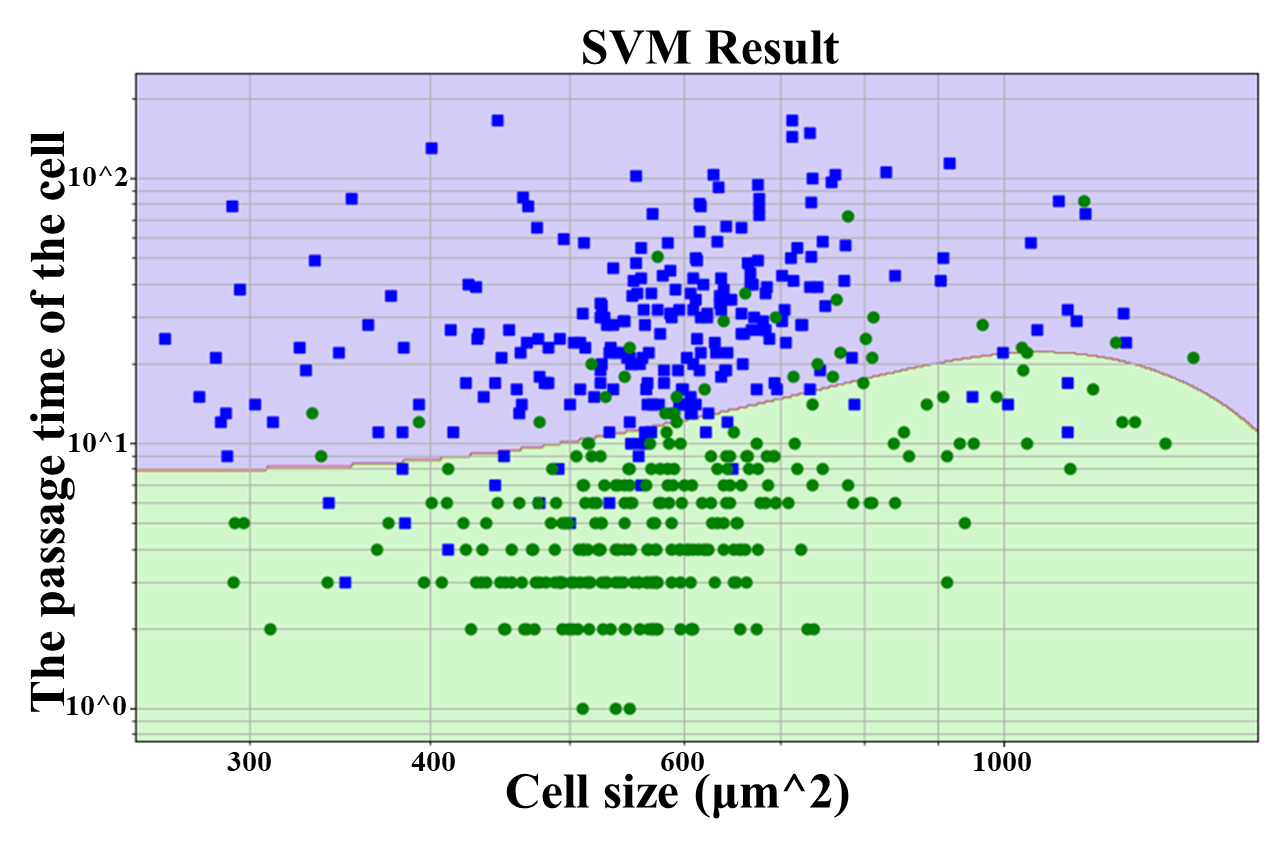


**Supplementary** **Figure 7 SVM for classifying MCF7 and MDA-MB-231 based on passage time and cell size.** Green dots represent data points of MDA-MB-231, while blue dots represent MCF7.


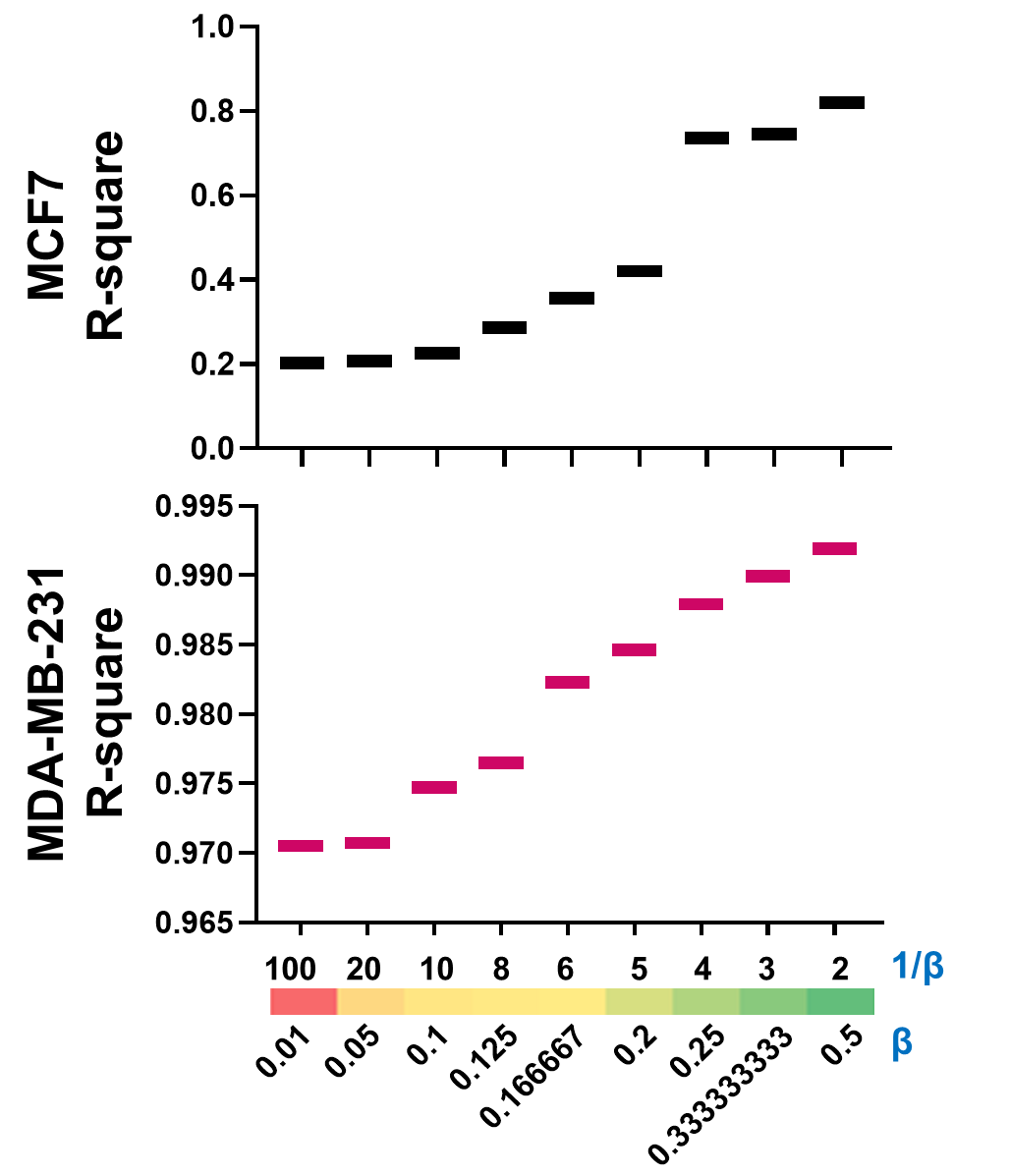


**Supplementary** **Figure 8 Evaluation of suitable power-law exponent β value for fitting** **mechanical model of cell rheology.** Different β values were applied to the mechanical model to evaluate the fitting outcome (R-squared).


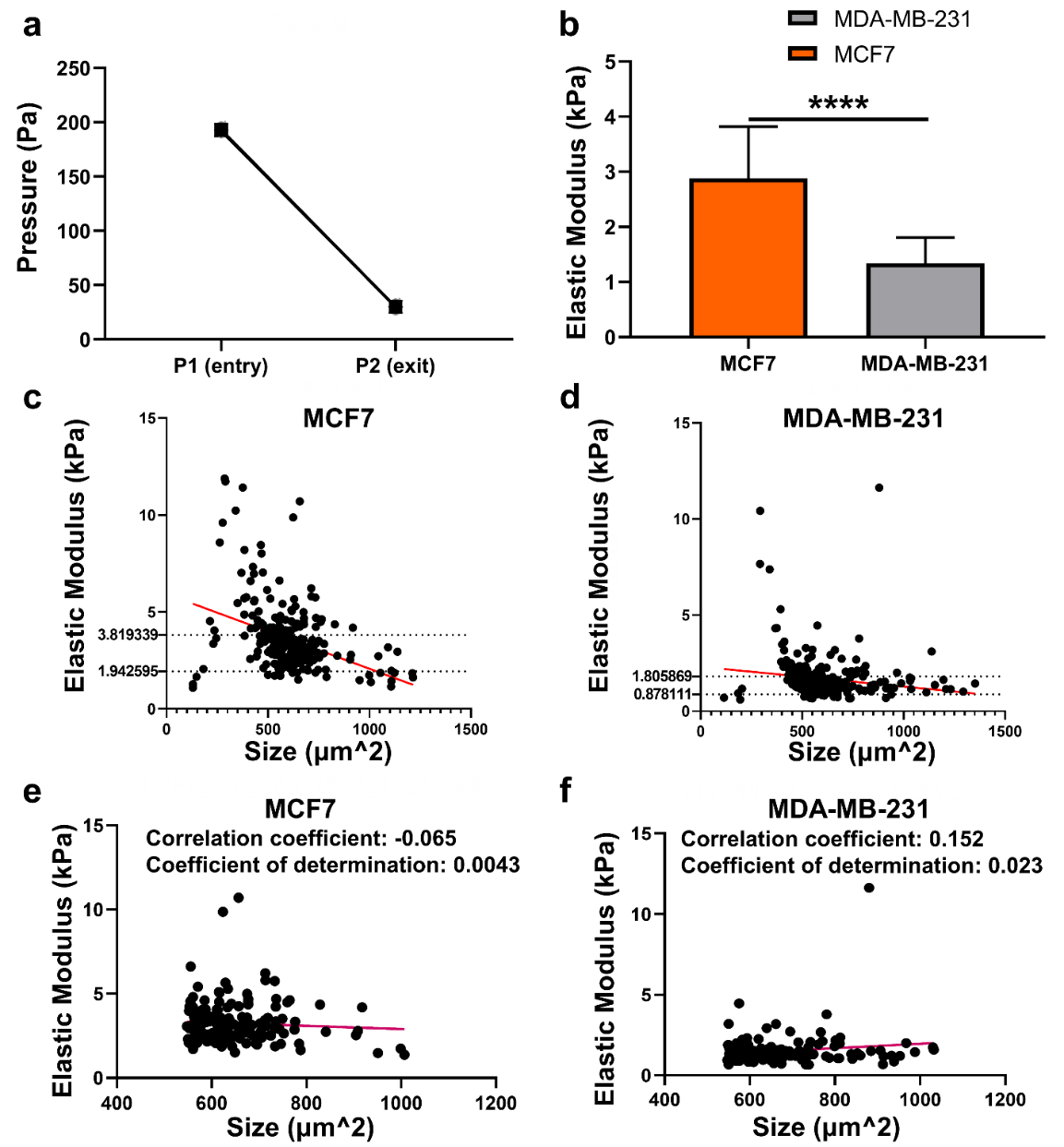


**Supplementary Figure 9 Evaluation of the effect of elastic modulus and size of MCF7 and MDA-MB-231. (a)** Pressure difference at the entries and the exits of microconstrictions on a cDC device. The line chart contains 36 lines representing the pressure difference of the 36 microconstrictions. The 36 lines overlapped due to their highly similar P1 and P2 values. **(b)** Calculated elastic modulus of MCF7 and MDA-MB-231. Statistical significance was calculated by a two-tailed Student’s t-test (P-value > 0.05: ns; 0.05: *; <0.01: **; <0.001: ***; <0.0001: ****). **(c)** Linear regression model demonstrating the relationship between cell sizes and calculated elastic modulus of MCF7. Dash lines represent the values of the mean elastic modulus of MCF7 ± standard deviation. **(d)** Linear regression model demonstrating the relationship between cell sizes and calculated elastic modulus of MDA-MB-231. Dash lines represent the mean elastic modulus values of MDA-MB-231 ± standard deviation. **(e)** Dot plot showing the data of MCF7 cells filtered by the determined optimal size range. Pearson correlation coefficient and coefficient of determination were shown on the plot. **(f)** Dot plot showing the data of MDA-MB-231 cells filtered by the determined optimal size range. Pearson correlation coefficient and coefficient of determination were shown on the plot.


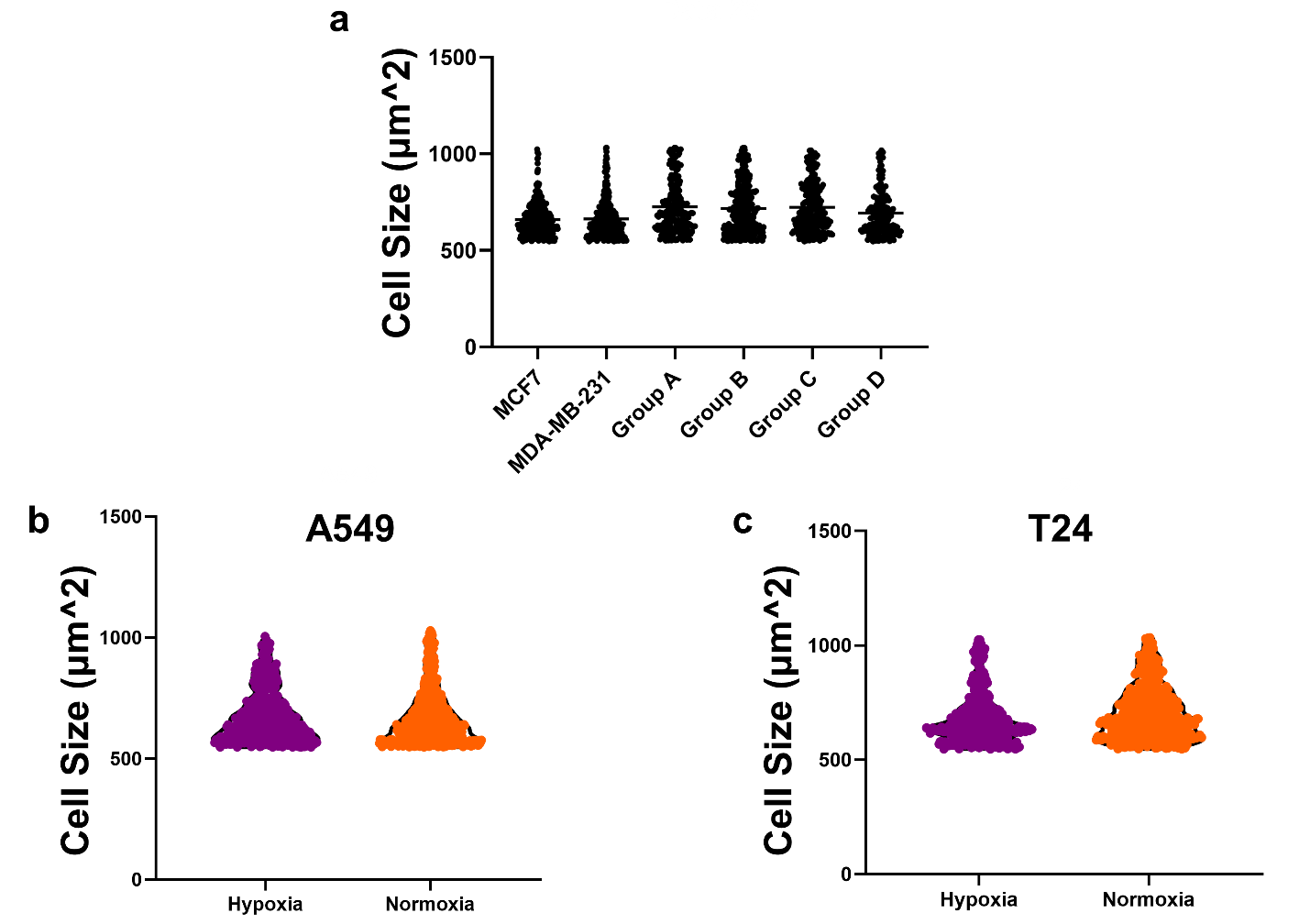


**Supplementary Figure 10 Size distribution of cancer cells involved in stiffness measurement. (a)** Size distribution of cells representing MCF7 only, MDA-MB-231 only, and mixed group A to D. **(b)** Size distribution of hypoxia- and normoxia-treated A549 cells. **(c)** Size distribution of hypoxia- and normoxia-treated T24 cells.


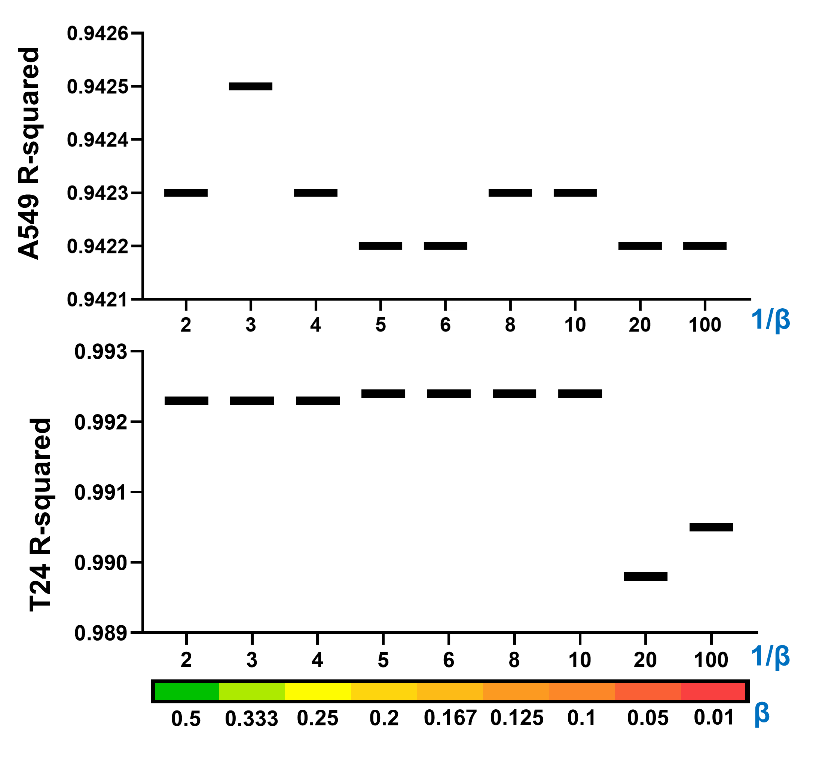


**Supplementary Figure 11 Evaluating power-law exponent β value for fitting data of A549 and T24.** Different β values were applied to the mechanical model to evaluate the fitting outcome (R-squared).


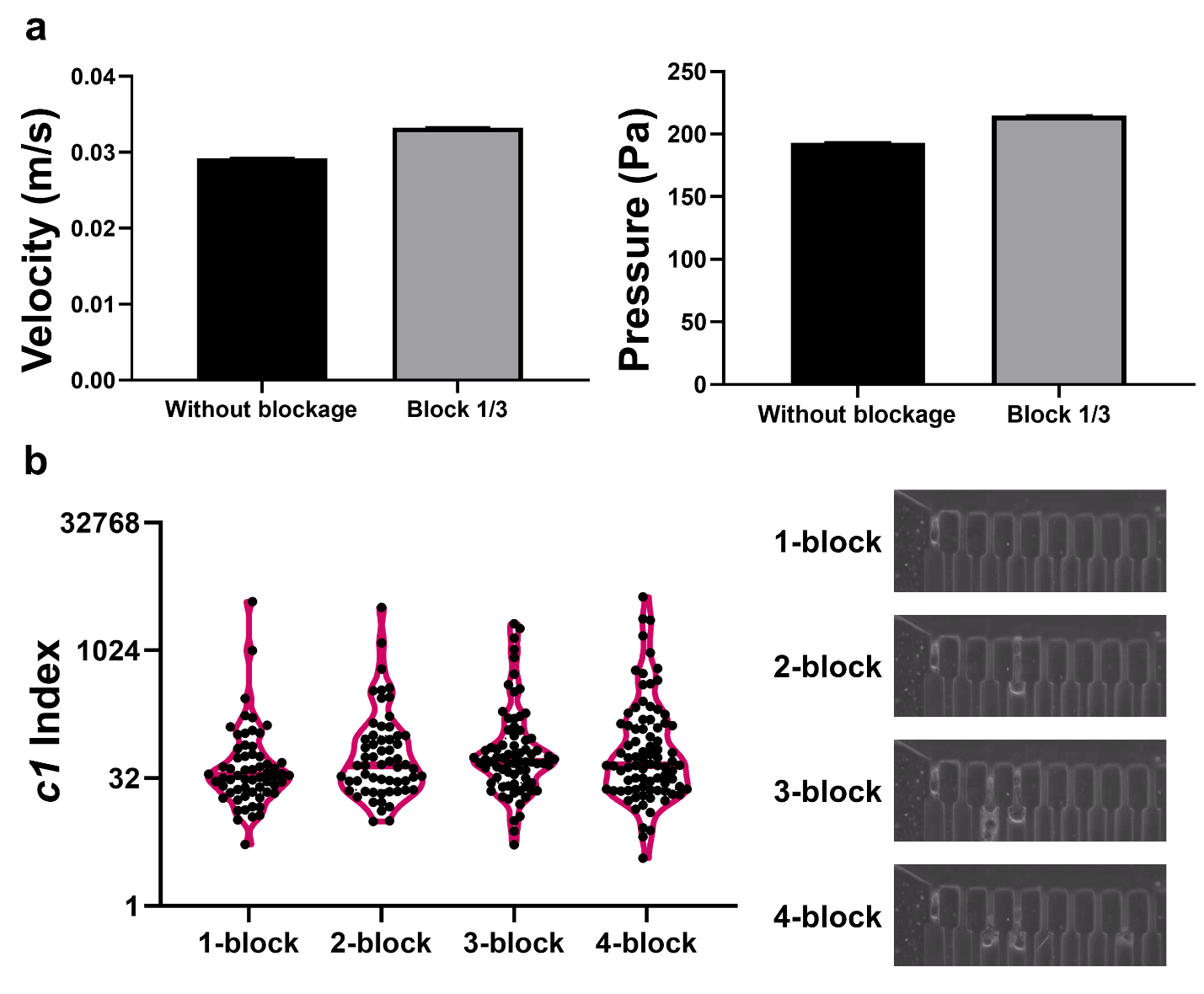


**Supplementary Figure 12 Investigation on microconstriction-related device fouling and their effects on measurements. (a)** Fluidic simulation for quantifying the effects on fluidic features at microconstrictions before and after fouling 1/3 of the microconstrictions. **(b)** Comparison of the c1 index of the same batch of cells measured on the same cDC device under different fouling statuses. Bladder cancer cell line T24 was applied for this test.

**References**

1 Bouchalova, P. & Bouchal, P. Current methods for studying metastatic potential of tumor cells. *Cancer Cell International* **22**, 394, doi:10.1186/s12935-022-02801-w (2022).

2 Surumbayeva, A. *et al.* Preparation of mouse pancreatic tumor for single-cell RNA sequencing and analysis of the data. *STAR protocols* **2**, 100989, doi:10.1016/j.xpro.2021.100989 (2021).

3 Xu, K. *et al.* Single-cell RNA sequencing reveals cell heterogeneity and transcriptome profile of breast cancer lymph node metastasis. *Oncogenesis* **10**, 66, doi:10.1038/s41389-021-00355-6 (2021).
